# Mapping gene flow between ancient hominins through demography-aware inference of the ancestral recombination graph

**DOI:** 10.1371/journal.pgen.1008895

**Published:** 2020-08-06

**Authors:** Melissa J. Hubisz, Amy L. Williams, Adam Siepel

**Affiliations:** 1 Department of Biological Statistics and Computational Biology, Cornell University, Ithaca, New York, United States of America; 2 Simons Center for Quantitative Biology, Cold Spring Harbor Laboratory, Cold Spring Harbor, New York, United States of America; Aarhus University, DENMARK

## Abstract

The sequencing of Neanderthal and Denisovan genomes has yielded many new insights about interbreeding events between extinct hominins and the ancestors of modern humans. While much attention has been paid to the relatively recent gene flow from Neanderthals and Denisovans into modern humans, other instances of introgression leave more subtle genomic evidence and have received less attention. Here, we present a major extension of the ARGweaver algorithm, called ARGweaver-D, which can infer local genetic relationships under a user-defined demographic model that includes population splits and migration events. This Bayesian algorithm probabilistically samples ancestral recombination graphs (ARGs) that specify not only tree topologies and branch lengths along the genome, but also indicate migrant lineages. The sampled ARGs can therefore be parsed to produce probabilities of introgression along the genome. We show that this method is well powered to detect the archaic migration into modern humans, even with only a few samples. We then show that the method can also detect introgressed regions stemming from older migration events, or from unsampled populations. We apply it to human, Neanderthal, and Denisovan genomes, looking for signatures of older proposed migration events, including ancient humans into Neanderthal, and unknown archaic hominins into Denisovans. We identify 3% of the Neanderthal genome that is putatively introgressed from ancient humans, and estimate that the gene flow occurred between 200-300kya. We find no convincing evidence that negative selection acted against these regions. Finally, we predict that 1% of the Denisovan genome was introgressed from an unsequenced, but highly diverged, archaic hominin ancestor. About 15% of these “super-archaic” regions—comprising at least about 4Mb—were, in turn, introgressed into modern humans and continue to exist in the genomes of people alive today.

## Author summary

We present ARGweaver-D, an extension of the ARGweaver algorithm which can be applied under a user-defined demographic model including population splits and migration events. Given genome sequence data from a collection of individuals across multiple closely related populations or subspecies, ARGweaver-D can infer trees describing the genetic relationships among these individuals at every location along the genome, conditional on the demographic model. Like ARGweaver, ARGweaver-D is a Bayesian method, sampling trees from the posterior distribution in order to account for uncertainty. Using simulations, we show that ARGweaver-D can successfully identify regions introgressed from Neanderthals and Denisovans into modern humans. It is also well-powered to detect introgressed regions stemming from older gene-flow events. We apply ARGweaver-D to the genomes of two Neanderthals, a Denisovan, and two African humans. We identify 3% of the Neanderthal genome which is likely derived from gene flow from ancient humans. We also identify about 1% of the Denisovan genome that may be traced to an unsequenced archaic hominin; 15% of these regions were subsequently passed to modern humans. We find no convincing evidence that selection acted against any of these introgressed regions.

## Introduction

It is now well-established that gene flow occurred among various ancient hominin groups over the past several hundred thousand years. The most well-studied example of archaic gene flow is the interbreeding that occurred when humans migrated out of Africa and came into contact with Neanderthals in Eurasia roughly 50,000 years ago [1, 2]. This event left a genetic legacy in modern humans that persists today; indeed, 1–3% of the DNA of living humans descended from non-African populations, such as Europeans or East Asians, can be traced to Neanderthals [3]. We also now know that an extinct sister group to the Neanderthals, the Denisovans, intermixed with early modern humans in Asia, leaving behind genomic fragments that comprise 2–4% of the DNA of modern Oceanian humans [4–6].

Many other admixture events have been proposed, creating a complex web of ancient hominin interactions across time and space. These events include gene flow between Neanderthals and Denisovans (Nea↔Den) [2, 7]; between Neanderthals and ancient humans who left Africa over 100 thousand years ago(Hum→Nea) [8]; between an unknown diverged or “super-archaic” hominin (possibly *Homo erectus*) and Denisovans (Sup→Den) [2, 9]; and between other unknown archaic hominins and various human populations in Africa (Sup→Afr) [10–12]. (In the above notation, used throughout this paper, the arrowheads indicate the inferred direction of gene flow, up to the limits of the data and inference method.).

As the network of interactions grows more complex, it becomes more difficult to test for gene flow or identify introgressed regions using standard methods [13]. In one prominent example, a positive value has been observed for a “*D* statistic” based on Neanderthals, Denisovans, African modern humans, and the chimpanzee reference genome [2], indicating an excess of allele sharing between Neanderthals and African humans, as compared with Denisovans and Africans. However, this observation potentially could be explained by gene flow between Neanderthals and Africans, boosting their allele sharing, or from super-archaic hominins into Denisovans, reducing Denisovan/African allele sharing. Notably, the *D* statistic is highest at sites where the derived allele is fixed or at high-frequency in Africans, implying that many of the excess shared alleles are quite old, and supporting the scenario of super-archaic introgression into Denisovans [2]. At the same time, however, many genomic windows with low Neanderthal-Africa divergence nevertheless have high Neanderthal-Denisovan divergence, which is best explained by Hum→Nea gene flow [8]. In this case, each hypothesis has support from multiple studies [8, 9, 14], suggesting that both the Hum→Nea and Sup→Den events likely occurred. But more generally, it can be difficult to resolve conflicting evidence of this kind using summary statistics alone.

Furthermore, even when there is strong evidence for the existence of gene flow, it remains challenging to identify particular introgressed genomic regions. This problem is considerably more difficult for the Sup→Den and Hum→Nea events than for the Nea→Hum or Den→Hum events, both because they are hypothesized to have occured much longer ago [2, 8], causing the introgressed haplotypes to be more broken up by recombination, and because no sequence is available for the super-archaic hominin. The small numbers of sequenced Neanderthal and Denisovan genomes are a further limitation. Current approaches for predicting introgressed regions, including the conditional random field (CRF) [3, 6] and the S* statistic [15, 16] (as well as the variant Sprime [17]), are not ideal for detecting these ancient events, having been optimized for the easier problem of identifying more recent introgression into humans. Furthermore, these methods only use a few summary statistics. When the genomic signal is more subtle, it may be necessary to incorporate all the data using a model-based method.

In this paper, we describe a powerful and highly general new method, called ARGweaver-D, that samples ancestral recombination graphs (ARGs) [18–20] conditional on a generic demographic model, including population divergence times, size changes, and migration events. After introducing ARGweaver-D, we present simulation studies showing it can successfully detect Nea→Hum introgression, even when using a limited number of genomes, and that it also has power for older migration events, including Hum→Nea, Sup→Den, and Sup→Afr events. Finally, we apply this method to modern-day Africans and ancient hominins, and characterize both new and previously reported cases of introgression between humans and archaic hominins.

## Results

### ARGweaver-D can sample ARGs conditional on an arbitrary demographic model

ARGweaver-D is a major extension of ARGweaver [21] that can infer ARGs conditional on a user-defined population model. This model can consist of an arbitrary number of present-day populations that share ancestry in the past, coalescing to a single panmictic population by the most ancestral discrete time point. Population sizes can be specified separately for each time interval in each population. Migration events between populations can also be added; they are assumed to occur instantaneously, with the time and probability defined by the user. Typically, a suitable demographic model for use with ARGweaver-D can be obtained from the literature or by applying a method such as ∂a∂i [22] or G-PhoCS [14] in a preprocessing step.

**Fig 1:**
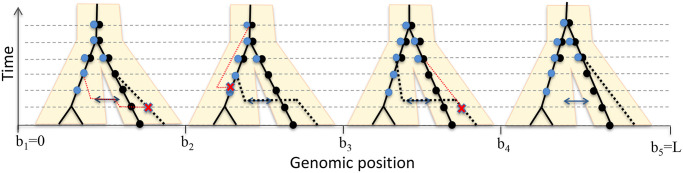
Illustration of the “threading” operation under a model with two populations and a single migration band. Horizontal dashed lines indicate time points for coalescence and recombination. User-specified migration and population divergence times are rounded to the nearest “half time-point”. Migration occurs instantaneously with a user-specified prior rate *p*_*M*_ (1% in this work). Here, one haploid lineage has been removed and is being rethreaded (dotted black line), while the other three (solid black lines) are held fixed. Dots on top of each lineage indicate potential coalescence points with the new branch, with black indicating a population path with no migration, and blue indicating a migrant population path. Recombination events (red Xs) occur immediately before positions *b*_2_, *b*_3_, and *b*_4_, with the dotted red line indicating recoalescence of the broken branch. Notice that the newly threaded lineage enters an introgressed state at position *b*_2_ and leaves it at *b*_4_.

As previously described [21], ARGweaver is a Markov chain Monte Carlo (MCMC) sampler, in which each iteration consists of removing a branch from every local tree in the ARG (“unthreading”), followed by the “threading” step, which resamples the coalescence points for the removed branches. This threading step is the core algorithmic operation in ARGweaver, and is accomplished using a hidden Markov model (HMM), in which the set of states at each site represents all possible coalescence points in the local tree. In ARGweaver (which assumes a single panmictic population), each of these states is defined by a branch and time. However, in ARGweaver-D, each state has a third property, which we call the “population path.” The population path represents the set of populations assigned to the new branch throughout its time span. The modified threading algorithm is illustrated and further described in Fig 1, and additional details are provided in S1 Text. ARGweaver-D is built into the ARGweaver source code, which is available at: http://github.com/CshlSiepelLab/argweaver.

After running ARGweaver-D, it is straightforward to identify predicted introgressed regions; they are encoded in each sampled ARG as lineages that follow a migration band. By examining the set of ARGs produced by the MCMC sampler, ARGweaver-D can compute posterior probabilities of introgression across the genome. As will be seen below, this computation can be done in a variety of ways—for example, as overall probabilities of migration anywhere in the tree, or probabilities of a specific sampled genome having an ancestral lineage that passes through a particular migration band. In addition, for a diploid individual, probabilities of heterozygous or homozygous introgression can be separately computed. Throughout this paper, we use a threshold of *p* ≥ 0.5 to define predicted introgressed regions, and compute total rates of called introgression for a diploid individual as an average across each haploid lineage.

### ARGweaver-D can accurately identify archaic introgression in modern humans

**Fig 2:**
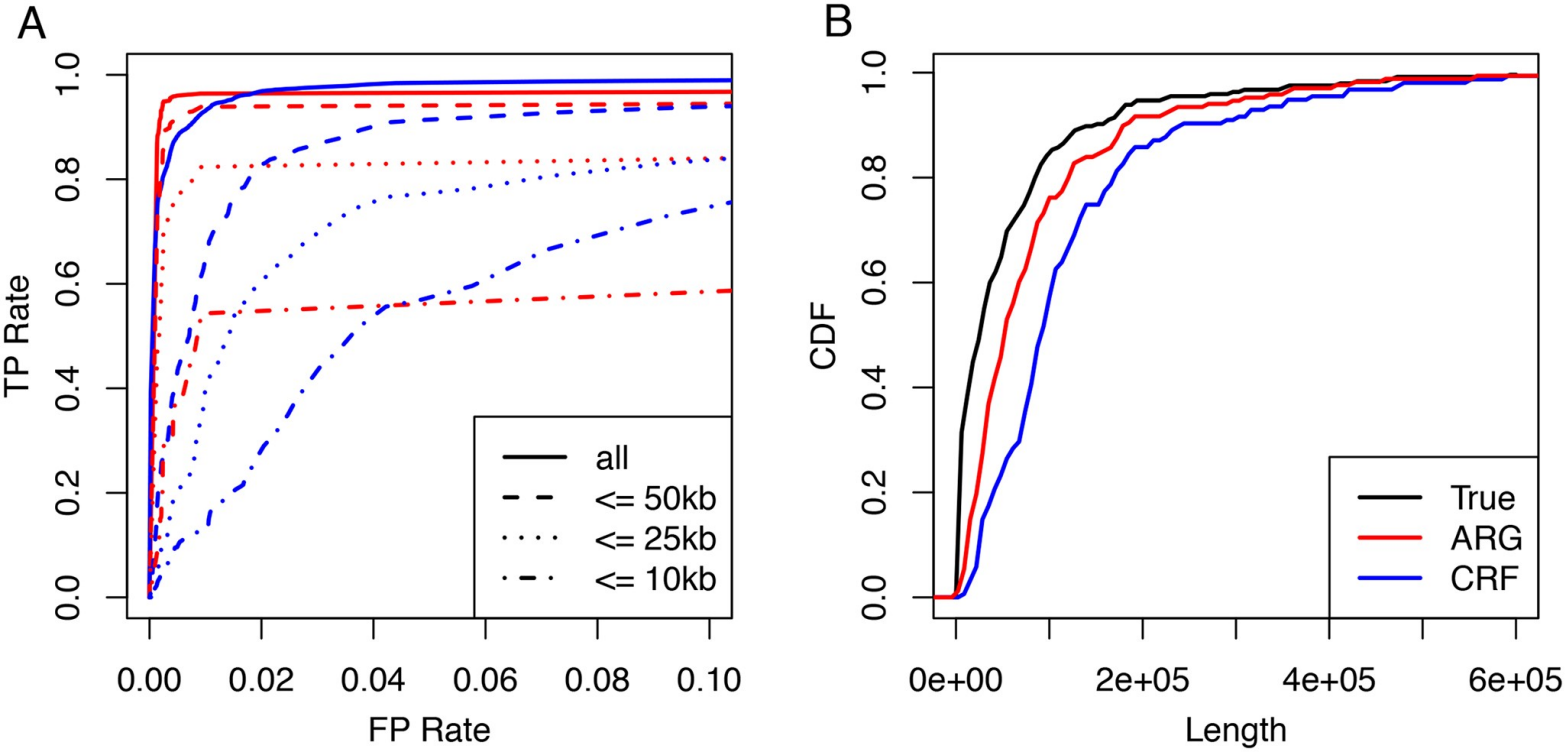
Performance on Nea→Hum simulations. **A**: Receiver operating characteristic (ROC) curves showing basewise performance of ARGweaver-D (red) and the CRF (blue) on simulated data. The two methods predicted introgression in the same simulated European individuals, but the CRF made use of the full reference panel (43 diploid Africans), whereas ARGweaver-D only used only two diploid Africans. Different line patterns correspond to different maximum segment lengths. **B**: Length distributions of real and predicted introgressed regions for data in panel A.

We first performed a set of simulations to assess the power and accuracy of ARGweaver-D in identifying Neanderthal introgression into modern humans. These simulations realistically mimic human and archaic demography, as well as variation in mutation and recombination rates (see Methods). We compared the performance with the CRF algorithm [3]; Fig 2 summarizes the results. Overall, ARGweaver-D has improved performance over the CRF, with improvements being subtle for long segments but becoming more pronounced for shorter segments. This gain in power occurs despite the fact that the CRF used a much larger panel of African samples than was used by ARGweaver-D. (CRF used 43 African individuals, but ARGweaver-D used only 2 to save computational cost; both methods used 2 diploid Neanderthals).

**Fig 3:**
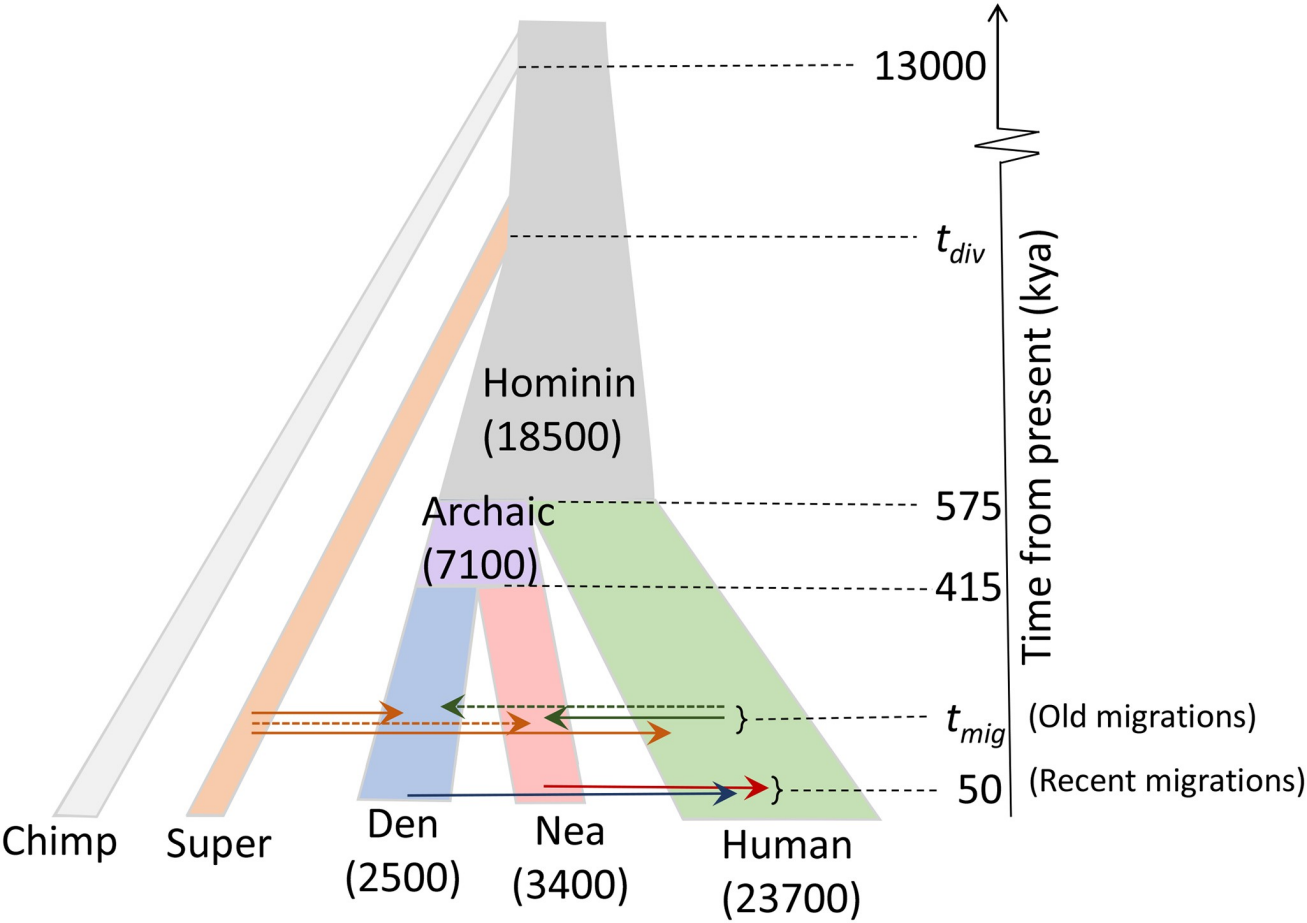
Population model assumed for inference using ARGweaver-D. Population sizes (constant per branch) are shown in parentheses. The model is invariant to the population sizes of the single-lineage chimpanzee and super-archaic hominin branches. Migration events are shown by arrows between populations; solid arrows are used for previously proposed events and dashed arrows for new events. All parameters except *t*_*mig*_ and *t*_*div*_ are held constant at the specified values.

**Fig 4:**
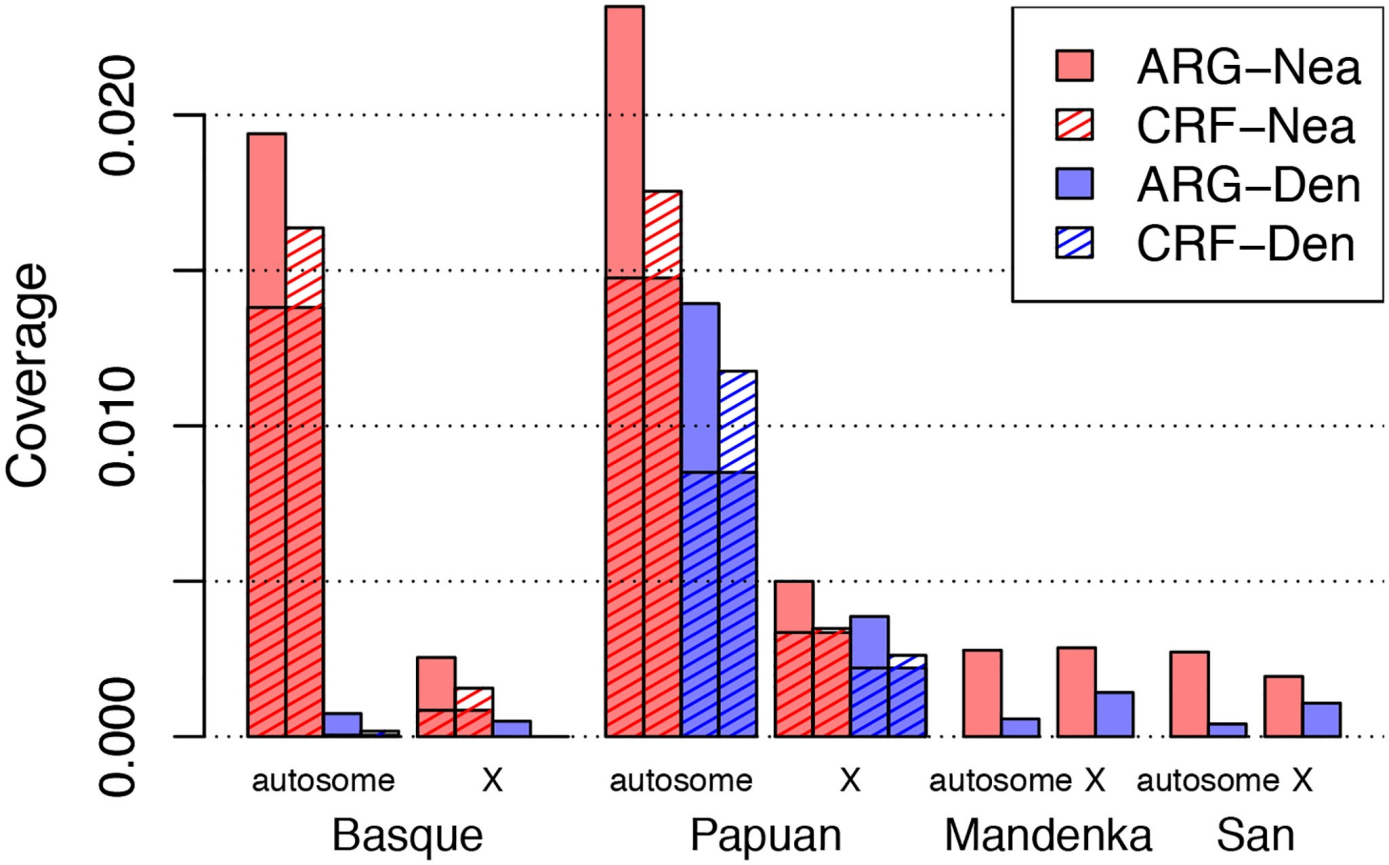
Average nucleotide coverage of predicted introgressed regions in four modern human individuals. Colors indicate ARGweaver-D predictions and stripes indicate CRF predictions; colors and stripes together indicate regions called by both methods. The CRF calls were only produced for non-African individuals, so for Mandenka and San, only ARGweaver-D results are shown. Genome sequences were from the Simons Genome Diversity Panel (SGDP).

Next, we predicted introgressed regions in two non-African human samples from the Simons Genome Diversity Panel (SGDP), a European (Basque) and a Papuan. The ARGweaver-D model used is illustrated in Fig 3; but only the “Recent migration” bands were included. We compared to calls from the CRF method, although it is important to note that the two methods were run with different data sets: ARGweaver-D again used many fewer African individuals (2) than CRF (43), but in this case ARGweaver-D used both the Altai and Vindija Neanderthal, whereas the CRF results were obtained with only the Altai. Because the Vindija Neanderthal is a better proxy for the introgressing Neanderthal, ARGweaver-D likely has better power to detect Neanderthal introgression in this comparison. The results are summarized in Fig 4. Overall, the two methods identify many overlapping regions, but each method also produces a substantial fraction not called by the other method (between 15-40%). Both methods show a strong depletion of introgression on the X chromosome, especially in the Basque individual.

Fig 4 highlights that more Neanderthal than Denisovan sequence is detected in the Papuan, despite that Papuans are expected to have a higher level of introgression from Denisovans compared to Neanderthal [23]. This observation can be explained by lower power to detect Denisovan introgression, due to the different levels of divergence between introgressing archaic individuals compared to sequenced archaic individuals; previous literature has shown that the sequenced Denisovan is highly diverged from the introgressing Denisovan [2], and that the Vindija Neanderthal is more closely related to the introgressing Neanderthal than the Altai Neanderthal [9]. In fact, this information is embedded in the ARGs and is reflected in the coalescence times between humans and archaic individuals in regions where these humans are introgressed. For example, the average coalescence time for introgressed lineages between Vindija and Papuan is 262kya; for Altai and Papuan is 326kya, and for Deniosvan and Papuan is 396ka. For the Basque individual, we also see a smaller average coalescence time with the Vindija (236kya) than the Altai (292kya).

Notably, ARGweaver-D calls nearly 0.5% introgression from the Neanderthal into each of the African individuals. These calls are likely explained by a combination of false positives and back-migration into Africa from Europe. However, another possibility is that some regions introgressed into Neanderthals from ancient humans [8] may be assigned the wrong direction by ARGweaver-D. With few samples, it can be difficult to determine the direction of migration between two sister populations. Indeed, when we simulate migration in both directions, but perform inference in ARGweaver-D using only a Nea→Hum migration band, we find that ∼8% of Hum→Nea bases are identified as Nea→Hum (See S1 Text). This difficulty in resolving directionality is our primary motivation for excluding non-African samples in our later analysis of older migration events (see next section).

### ARGweaver-D can detect older introgression events

We next carried out a series of simulations to assess ARGweaver-D’s power to detect more ancient introgression events. For this purpose, we simulated the modern human samples using a model of African human population history, and as such did not include the migration from Neanderthals or Denisovans into non-African humans. These simulations included three migration events: one from modern humans into Neanderthals (Hum→Nea), one from a “super-archaic” unsampled hominin into Denisovans (Sup→Den), and one from the super-archaic hominin into Africans (Sup→Afr). (Note that although both the Sup→Afr and Sup→Den events are simulated from the same super-archaic population, they are meant to represent introgression from any unsampled, diverged hominin population, not necessarily the same one.) These simulations included many realistic features: ancient sampling dates for the archaic hominins, variation in mutation and recombination rates, randomized phase, and levels of missing data modeled after the SGDP and ancient genomes that we use for analysis (see Methods). Each set of simulations contained all three types of migration, and ARGweaver-D was applied with multiple migration bands, with the goal of detecting all migration events in a single run.

We analyzed these data sets with ARGweaver-D using the model depicted in Fig 3, but including only the “old migration” bands. As we do not have good prior estimates for the migration time (*t*_*mig*_) or super-archaic divergence time (*t*_*div*_), we tried four values of *t*_*mig*_ (50kya, 150kya, 250kya, 350kya) and two values of *t*_*div*_ (1Mya, 1.5Mya). We generated data sets under all 8 combinations of *t*_*mig*_ and *t*_*div*_, and then analyzed each data set with ARGweaver-D under all 8 models, in order to assess the effects of model misspecification on the inference.

**Fig 5:**
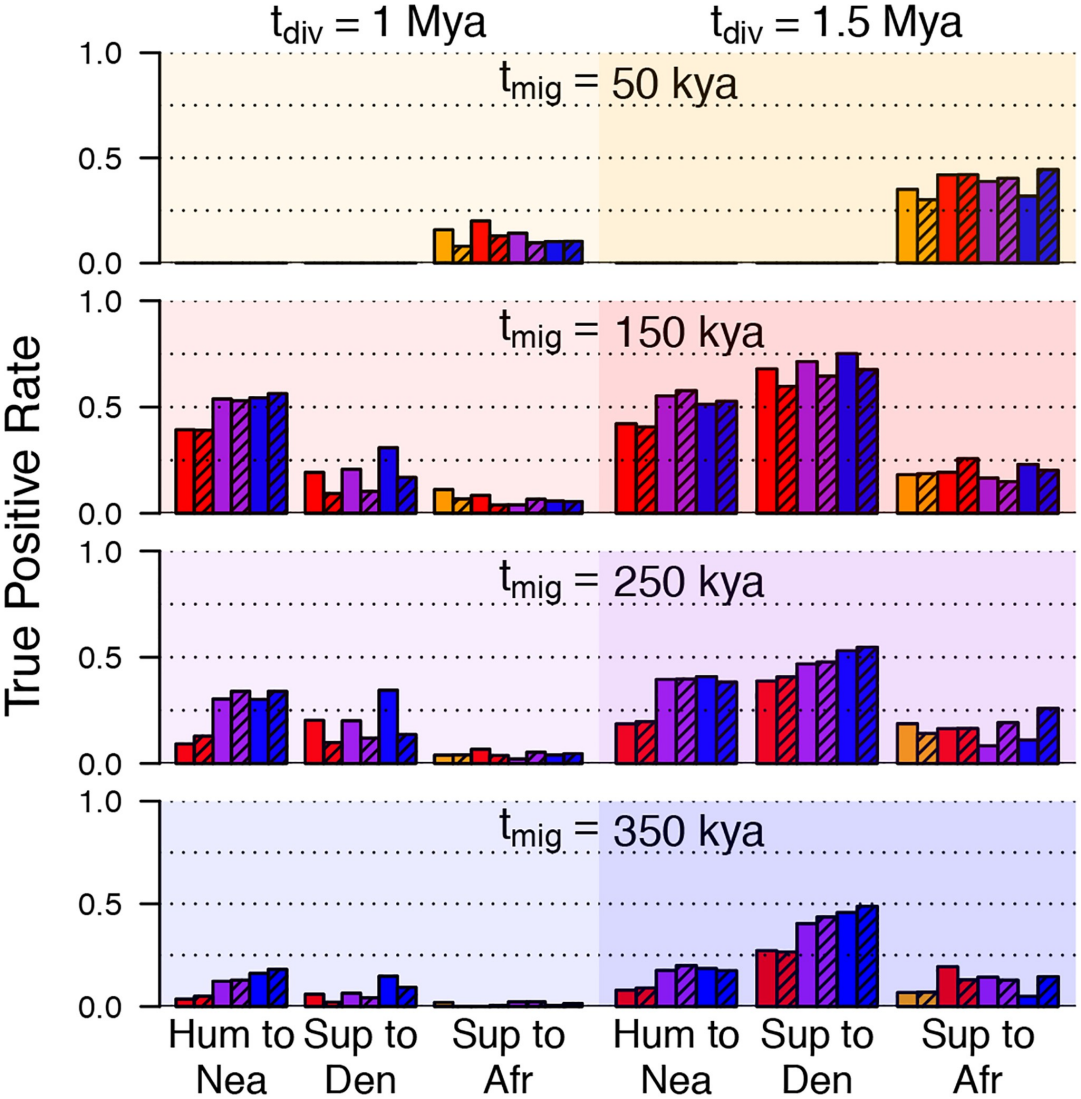
Simulation results. Each panel represents a set of simulations generated with a different value of *t*_*mig*_ (rows) and *t*_*div*_ (columns). Within each panel, each bar gives the basewise true positive rate for a particular migration event, using a posterior probability threshold of 0.5. The color of each bar represents the value of *t*_*mig*_ assumed for the inference model (orange = 50kya, red = 150kya, purple = 250kya, blue = 350kya). Shaded bars represent an assumption of *t*_*div*_ = 1.5Mya for inference, whereas solid bars represent *t*_*div*_ = 1.0Mya. Because the archaic hominin fossils are older than 50kya, results for *t*_*mig*_ = 50kya (top) are only applicable for introgression into humans.

We find that the power to detect super-archaic introgression is clearly higher when the divergence is higher (*t*_*div*_ = 1.5Mya), but, as expected, the choice of *t*_*div*_ does not affect the power to detect Hum→Nea introgression (Fig 5). In addition, for all events, we find that power decreases as the true migration time increases (from top to bottom in Fig 5).

Looking at each group of bars in Fig 5 shows the results on the same simulated data, using different parameters in the ARGweaver-D model. The blue and purple bars tend to be higher, showing that power is often better when an older migration time is used in the model, even when the true migration time is recent. Similarly, power is often better for detecting super-archaic introgression when *t*_*div*_ is set to 1Mya (solid), rather than 1.5Mya (striped), in the ARGweaver-D model. Overall, ARGweaver-D has reasonably good power to detect super-archaic introgression when the divergence time is old, but power is more limited as the divergence time decreases. The power to detect Sup→Afr is always lower than the power to detect Sup→Den, as the African population size is much larger, making introgression more difficult to distinguish from incomplete lineage sorting. For the Hum→Nea event, we have around 50% power if the migration time is 150kya, and around 30% power when it is 250kya.

False positive rates were < 1% at a posterior probability threshold of 0.5 (S1 Fig). In addition, two additional migration bands in the ARGweaver-D model served as controls for false positive predictions: one from the super-archaic population to Neanderthal (Sup→Nea), and another from humans into Denisova (Hum→Den). Events in these bands were also called at < 1% for all models. In addition, the rate of mis-classification of migration type was very low for all event types (S2 Fig). In particular, the model can easily distinguish between Hum→Nea and Sup→Den events, despite that both produce similar *D* statistics [8, 9].

Notably, the simulated data sets were generated with a human recombination map, but the ARGweaver-D model assumed a simple constant recombination rate (S1 Text). We observe somewhat better performance when ARGweaver-D uses the true recombination map, but it is unrealistic to assume the true map is known for the archaic hominins. In addition, we find that the performance of the method does not improve as more African samples are added, so we focus here on an analysis with two African samples only (four haploid genomes). In a separate simulation study, we find that the method is reasonably robust to errors in the assumed population size, although the false positive rate does increase if the population size of the population receiving migration is underestimated by more than 20%, with FP rates approaching 5% for Hum→Nea when the assumed Nenderthal population size is 25% of the true value. Further details of these simulation studies are provided in S1 Text.

### Deep introgression results

Having demonstrated reasonable power and accuracy in a simulation setting, we turned to an analysis of real modern and archaic human genomes. Our goals for this study were to identify and characterize introgressed regions from previously proposed migration events, as well as to look for evidence for new migration events, perhaps not detectable by other methods. Our data set consisted of two Africans from the SGDP [24], two Neanderthals [2, 9], the Denisovan [4], and a chimpanzee outgroup. For inference, we again assumed the demography illustrated in Fig 3, considering the old migration events only. We focus here on the model with *t*_*mig*_ = 250kya and *t*_*div*_ = 1Mya, because this model seemed to result in high power in all our simulation scenarios, and because our results suggest that it may be the most realistic (as discussed below). The results using other models are consistent with those presented here (see S1 Text).

**Fig 6:**
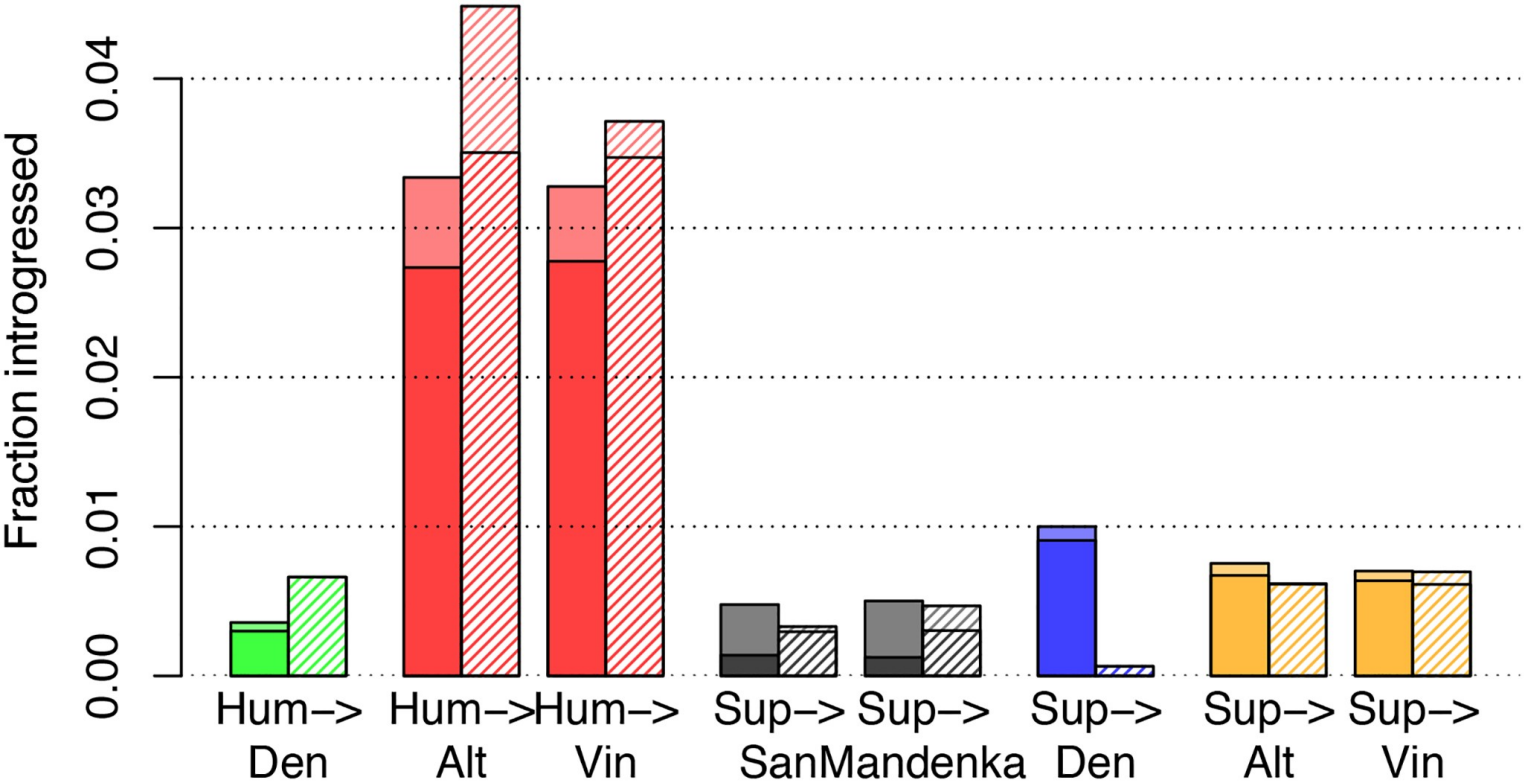
Genome-wide coverage of predicted ancient introgression. Each bar shows total average coverage for a haploid genome, with darker shading (at bottom) representing homozygous calls. Solid bars are for autosomes, and striped bars for chromosome X. Predictions were based on a posterior probability cutoff of 0.5.

Overall, we find that Hum→Nea regions are called most frequently, at a rate of ∼3% in both the Altai and Vindija Neanderthal (Fig 6; see also S3 Fig). This number is almost certainly an underestimate, given that the true positive rate for this model was estimated at 30–55%. By contrast, only ∼0.37% of regions are classified as Hum→Den. As no previous study has found evidence for Hum→Den migration, this migration band serves as a control, supporting our false positive rate estimate of 0.41% from simulations.

As noted, there is a well-known depletion on the X chromosome of archaic introgression into humans. By contrast, we observe high coverage of Hum→Nea introgression on the X chromosome for both the Altai and Vindija samples. Indeed, the coverage is somewhat higher on the X chromosome than the autosomes. However, this difference is likely due in large part to increased power on the X; simulations suggest that power will be ∼20% higher for this event when effective population sizes are multiplied by 0.75 (S4 Fig). Nevertheless, we observe considerable variation in detected introgression across the chromosomes, and several autosomal chromosomes have higher predicted coverage than the X, including chromosomes 1, 6, 21, and 22 (S3 Fig).

Although the Vindija sample is younger by 70kya than the Altai sample [9], it shows no depletion of human ancestry on the autosomes, suggesting that negative selection did not cause a significant loss of human introgressed regions during that interval. However, some individual chromosomes do show decreases in coverage from Altai to Vindija, with the largest drop on the X chromosome (S3 Fig).

Other migration events are detected at lower levels. We identify 1% of the Denisovan genome as introgressed from a super-archaic hominin—roughly double the estimated false positive rate (0.49%) for this event. Our apparent weak power for these events (another group has estimated ∼6% introgression [9]) suggests that the super-archaic divergence may have been somewhat recent (perhaps closer to 1Mya than 1.5Mya). Still, this analysis resulted in 27Mb of sequence that may represent a partial genome sequence from a previously unsequenced archaic hominin. In addition, ARGweaver-D predicted that a small fraction of the Neanderthal genomes is introgressed from a super-archaic hominin (0.75% for Altai and 0.70% for Vindija), an event that has not been previously hypothesized. However, these fractions only slightly exceed the estimated false positive rate (0.65%), so these results are likely dominated by spurious predictions.

The Sup→Den events (and perhaps Sup→Nea events) raise the possibility that super-archaic-derived sequences could have been passed, in turn, to modern humans through subsequent Den→Hum (or Nea→Hum) migration events. To explore this possibility, we intersected the predicted regions with introgression predictions in modern humans across the full SGDP data set (details in S1 Text). We found that most Sup→Den and Sup→Nea regions have higher-than-expected divergence to the Denisovans and Neanderthals (respectively) across all humans, and not just the two African humans analyzed by ARGweaver-D. In addition, 15% of the Sup→Den regions overlap with sequence introgressed into Asian and Oceanian individuals from Denisovans, and many of these regions also contain a high number of variants consistent with super-archaic introgression. We also observe that 35% of the Sup→Nea regions are introgressed in at least one modern-day non-African human. Notably, one region of hg19 (chr6:8450001-8563749) appears to be Neanderthal-introgressed and also overlaps a Sup→Nea region. A complete list of Sup→Den and Sup→Nea regions that overlap human introgressed regions, and the genes that fall in these regions, is available in S1 and S2 Tables.

We sought to obtain an improved estimate of the timing of migration the Hum→Nea event using the predicted introgressed regions. Initially, we attempted to gain information about timing from the segment lengths. However, we found that there is strong ascertainment bias towards finding longer regions, so that the length distributions are highly overlapping for different migration times (S1 Text). Instead, we turned to the frequency spectrum of introgressed regions, which provides a more robust signal. The older the migration, the more likely that an introgressed region has drifted to high frequency and is shared across the sampled individuals. For the Hum→Nea event, we found that 37% of our regions are inferred as “doubly homozygous” (that is, introgressed across all four Neanderthal lineages). This faction is close to what we observe in regions predicted from our simulations with migration at 250kya (38%), whereas simulations with migration at 150kya and 350kya had substantially different doubly-homozygous rates of 10% and 55%, respectively. To obtain a more precise estimate, we performed additional simulations with values of *t*_*mig*_ = 200, 225, 275, and 300kya, and compared the frequency spectrum of introgressed regions after ascertainment using ARGweaver-D. Overall, we find that the divergence time cannot be pinpointed precisely by this method, but it can be fairly confidently bounded at 200kya < *t*_*mig*_ < 300kya (S5 Fig). The same approach suggests that *t*_*mig*_ > 225kya for the for the Sup→Den event (S6 Fig).

#### Data release and browser tracks

**Fig 7:**
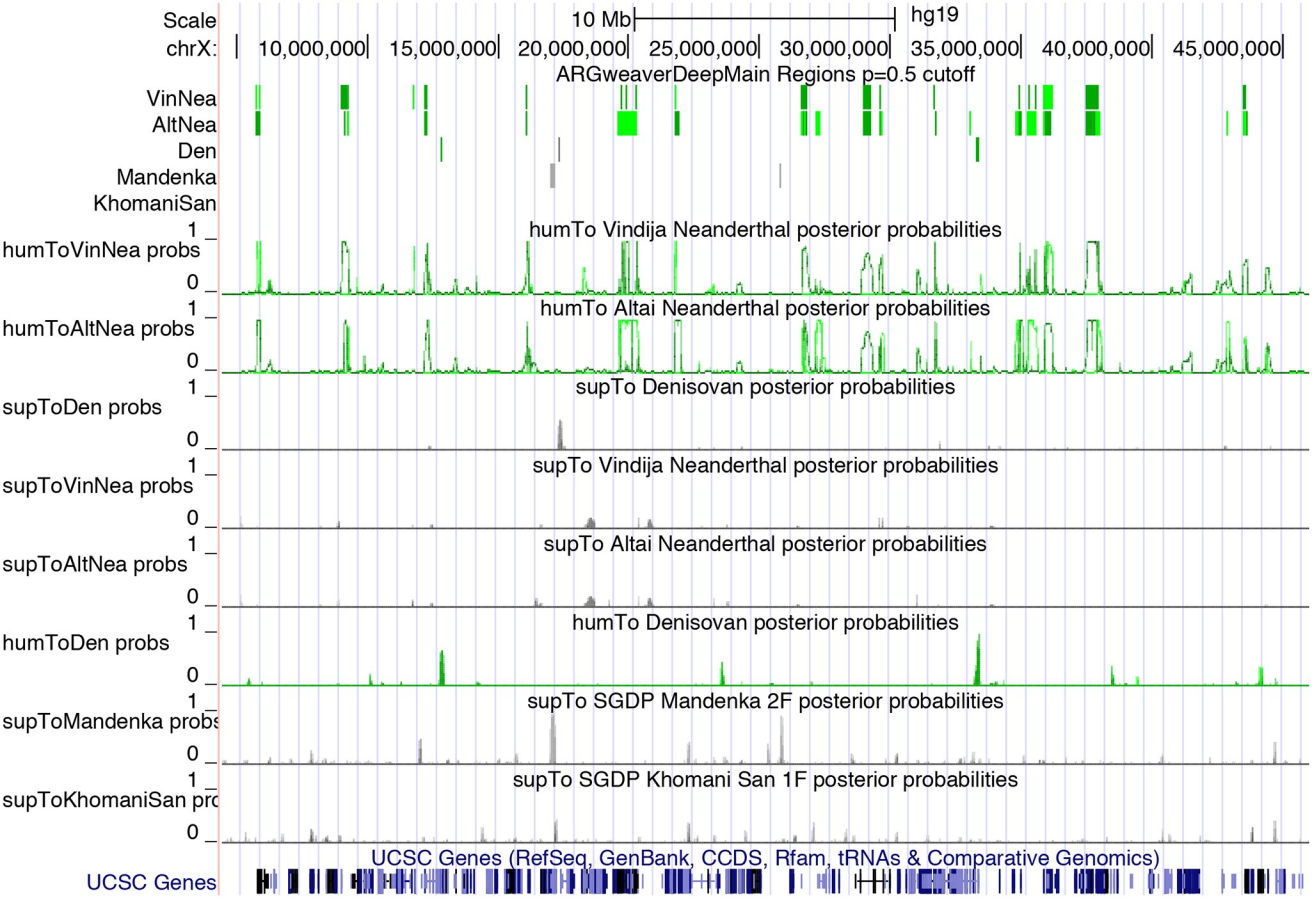
Introgression results for a ∼40Mb region of chromosome X displayed in the UCSC Genome browser. The tracks at top show predicted introgressed regions, with green indicating introgression from humans, and gray indicating introgression from a super-archaic hominin. Darker colors are used for homozygous introgression. The tracks below indicate the posterior probabilities for each type of introgression into each individual.

**Fig 8:**
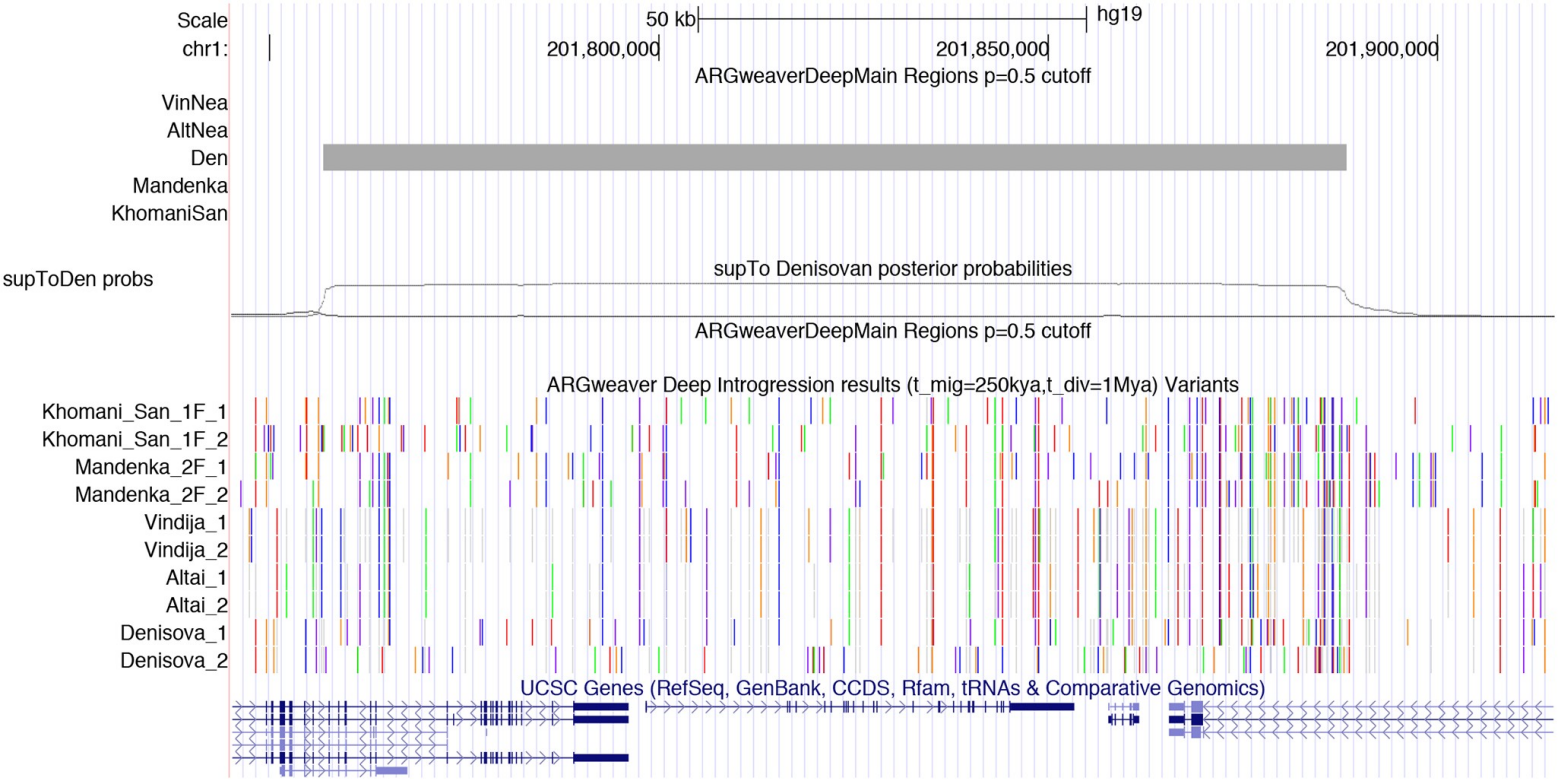
A region with predicted heterozygous Sup→Den introgression. A ∼170kb region on human chromosome 1 is shown in the UCSC Genome Browser. The first two tracks show the predicted regions and posterior probabilties as in Fig 7, except only the supToDen probabilities are shown. The next track shows the variants observed in this region that are used in the ARGweaver-D analysis, with alternating colors indicating variant alleles. Notice that, outside of the introgressed region, the Denisovan is generally homozygous and shares variants with Africans and Neanderthals; but within the region, the Denisova_2 haplotype has many singleton variants, whereas Denisova_1 continues to share many variants with Neanderthals and Africans. When chimpanzee alignments are available, the non-chimp allele is colored; otherwise the minor allele is colored. No color indicates that a haplotype has the chimpanzee or major allele, or missing data. The final phasing sampled by the ARGweaver-D algorithm was used.

Our predictions and posterior probabilities can be viewed as a track hub on the UCSC Genome Browser [25], using the URL: http://compgen.cshl.edu/ARGweaver/introgressionHub/hub.txt. The raw results can be found in the sub-directory: http://compgen.cshl.edu/ARGweaver/introgressionHub/files. Fig 7 shows a large region of chromosome X as viewed on the browser, with a set of tracks showing called regions, and another showing posterior probabilities. Fig 8 shows a zoomed-in region with a Sup→Den prediction, and S7 Fig shows an example Hum→Nea region. When zoomed in, there is a track showing the patterns of variation in all the individuals used for analysis, with haplotype phasing sampled from ARGweaver-D.

#### Functional analysis of introgressed regions

Because some of our observations suggested an absence of selection against the Hum→Nea regions, we searched for other signals that might hint at possible functional consequences of this introgression event. We first looked at four 10Mb introgression “deserts,” where the rate of both Nea→Hum and Den→Hum introgression is < 1/1000 [6]. We observed fairly high coverage of Hum→Nea introgression in these deserts (Table 1), suggesting that the bias against introgression is unidirectional. For two of the deserts, the Hum→Nea coverage is quite high, especially in the Altai Neanderthal. Notably, the third region overlaps the *FOXP2* gene, which contains two human-chimp substitutions that have been implicated in human speech [26, 27], although the Hum→Nea introgressed region is upstream of these substitutions (S8 Fig).

**Table 1:** Amounts of Hum→Nea introgression in deserts of Nea→Hum and Den→Hum introgression.

Location (hg19)	cov Altai	cov Vindija	num Altai	num Vindija
chr1:99-112 Mb	0.097	0.024	4	3
chr3:78-90 Mb	0.101	0.078	6	5
chr7:108-128 Mb	0.023	0.031	4	5
chr13:49-61 Mb	0.029	0.048	6	5

The columns “cov Altai” and “cov Vindija” show the coverage of Hum→Nea within the given region on each Neanderthal; “num Altai” and “num Vindija” show the number of introgressed regions > 50kb. The genome-wide average coverages for Altai and Vindija are 0.034 and 0.033, respectively.

We further examined a broader collection of deserts of Nea→Hum ancestry for evidence of depletion for introgression in the opposite direction. Based on the CRF regions, we identified 30 regions of at least 10Mb that meet the criteria for “deserts”. However, by several measures—including coverage of Hum→Nea, number of elements, and change in coverage between the Altai and Vindija Neanderthals—these deserts are not significantly different from a set of randomly chosen genomic regions matched for size (S9 Fig).

Finally, we checked for enrichments or depletions of various functional elements in our introgressed segments, relative to the expectation under a random distribution across the genome. The interpretation of these numbers is difficult, as local genomic factors (such as effective population size, mutation and recombination rates) significantly affect ARGweaver-D’s power to detect introgressed regions. Nevertheless, we find that the enrichment of functional regions (such as CDSs, promoters, and UTRs) tends to be higher in the Altai than the Vindija Neanderthal, which is the opposite pattern expected from negative selection (since the Vindija Neanderthal’s fossil is much more recent). Further enrichment results are detailed in S1 Text.

## Discussion

In this article, we present a new method, called ARGweaver-D, for sampling ancestral recombination graphs (ARGs) under an arbitrary demographic model, and show that this method is particularly powerful for identifying introgressed regions. Like our previous ARGweaver method, ARGweaver-D is limited to use with relatively small sample sizes (up to about 100 haploid genomes) owing to its considerable computational demands, but even in this setting it shows excellent power in the detection of introgression, particularly older events. In addition, ARGweaver-D has several other benefits over alternative methods for detection of gene flow. For example, it does not require a reference panel of non-introgressed individuals, and it can simultaneously identify introgression stemming from multiple migration events, as well as from both sampled or unsampled populations. In addition, ARGweaver-D does not rely on summary statistics, but uses a model of coalescence and recombination to generate local gene trees that are most consistent with the observed patterns of variation, even for unphased genomes. By incorporating all this information simultaneously, it can successfully distinguish migration from incomplete lineage sorting, and tease apart different migration events that produce similar *D* statistics (such as Sup→Den and Hum→Nea). The ARGweaver-D code is freely available and can be applied to any similar collection of DNA sequences and user-defined demographic scenario.

While we have focused in this article on introgression, ARGweaver-D is a fully general method for demography-aware ARG inference and could potentially have many other applications. The ARGs sampled by ARGweaver-D—by reflecting constraints on coalescence consistent with isolation and migration scenarios, as well as differences in effective size across populations—are likely to be considerably more accurate than those inferred by ARGweaver, which assumes a panmictic population of constant size. These differences may be particularly important in cases in which populations have dramatically different sizes (as with modern and archaic humans) or in cases of complete or near-complete population isolation. These improvements could be relevant in many applications of ARG inference, including the detection of sequences under selection or estimation of allele ages [21]. Moreover, ARGweaver-D can be useful in evaluating the relative likelihoods of alternative demographic models (although formal model testing in this framework remains a challenging and unsolved problem). Finally, it is worth emphasizing that in multi-population settings with limited migration, ARGweaver-D can be considerably more efficient than ARGweaver, because constraints on permissible coalescence events result in a substantial reduction in the state space of the HMM used for the core “threading” operation.

A recently published method, dical-admix [28], is similar to ARGweaver-D in that it is designed to accommodate generic demographic models and considers the full haplotype structure of the input sequences. This method appears to be quite powerful, but it does assume that there are only a few admixed individuals, and that other genomes are “trunk” lineages that help define the haplotype structure of their respective populations. Therefore, unlike ARGweaver-D, dical-admix cannot infer admixture from an unsampled population, nor is it designed to work when all individuals have some degree of admixed ancestry. Additionally, ARGweaver-D has the advantage of allowing for unphased genomes, which is important since there are currently too few Neanderthal or Denisovan samples to permit reliable phasing. In addition, two methods have recently been published that permit approximate gene-tree or ARG inference on much larger scales (with up to hundreds of thousands of samples) [29, 30] but, as yet, these methods do not consider the constraints of a demographic model or allow for uncertainty in the ARG given the data. More work would be needed to optimize them for use in detecting introgression events.

By applying ARGweaver-D to modern and archaic hominins, we confirmed that a substantial proportion of the Neanderthal genome consists of regions introgressed from ancient humans. While we identified only 3% of the Neanderthal genome as introgressed, a rough extrapolation based on our estimated rates of true and false positives suggests that the true fraction is ∼7% (S1 Text). Thus, the Neanderthal genome likely includes a larger contribution from ancient humans than modern non-African human genomes include from Neanderthal introgression.

Our follow-up analysis based on the frequencies of introgressed elements among the two diploid Neanderthal genomes suggests that the Hum→Nea gene flow occurred roughly between 200 and 300kya, within the limits of accuracy imposed by our assumed demographic model, mutation rates, and generation time. As previously noted [8, 9], because contact between modern humans and Neanderthals most likely took place in Eurasia, this timeline appears to be inconsistent with a genetic exchange involving the direct ancestors of most present-day Eurasians, who migrated out of Africa ∼50kya. Instead, our timeline suggests an earlier migration, occurring at least 200kya. Notably, orthogonal lines of evidence now support the possibility of one or more such early migrations out of Africa. For example, mitochondrial DNA from Neanderthals has much lower divergence than expected from human mtDNA (with the exception of the Sima de los Huesos samples, which cluster with Denisovans) [31]. This observation suggests that most Neanderthal mtDNA is introgressed from ancient humans. A recent study [32] bounded this introgression event at ≥ 270kya, in rough agreement with our estimated timeline, under the assumption that the recently analyzed Hohlenstein-Stadel sample and all sequenced Late Pleistocene Neanderthals share a common mtDNA origin outside of Africa [31]. Beyond this genetic evidence, there have also been discoveries of ancient fossils with human characteristics outside of Africa, including a 180kya jawbone from Misliya Cave in Israel [33], and a 210kya fossil from Apidima Cave in southern Greece with human features [34]. These findings suggest that early modern humans were present on the Eurasian continent at roughly the time at which Hum→Nea gene flow is estimated to have occurred. These early migrating humans may later have gone extinct, leaving a genetic trace only in introgressed segments in Neanderthals.

Aside from the issue of timing is the question of the possible functional impact of the Hum→Nea introgression. This question has proved challenging due to the myriad ascertainment biases—known and unknown—that affect the power to detect introgressed regions. Even in the case of Nea→Hum migration, for which power to detect introgression is high, earlier claims of depletion near genes, as well as decreasing levels of introgression over time, have been recently called into question [13, 28]. The strongest remaining pieces of evidence for negative selection against Nea→Hum introgression are the depletion on the X chromosome and in several other genomic deserts. But for the Hum→Nea event, we see no depletion on the X, and while we had too few samples to detect deserts across Neanderthals, we did confirm that previously identified Nea→Hum deserts are not depleted for Hum→Nea introgression. We do see a slight decrease in Hum→Nea introgression on the X chromosome in the Vindija Neanderthal compared to the Altai, which could be explained by weak negative selection removing some introgressed regions in the ∼70ky interval between the ages of these fossils. An interesting question is whether the observed absence of negative selection reflects healthy variation introduced by human introgression into the Neanderthal genome, or a Neanderthal population that was too small for efficient removal of deleterious variants. With additional archaic samples, it may be possible to address these questions.

ARGweaver-D also identified 1% of the Denisovan genome as introgressed from a super-archaic hominin. Previous studies have attributed roughly 6% of the Denisovan genome to this event [9], but ours is the first study to identify specific introgressed regions. Our apparent weak power to detect these regions suggests that the introgressing population was not too highly diverged from other hominins; according to our simulations, the observed sensitivity is more consistent with a divergence time of 1Mya than 1.5Mya. Still, we identify 27Mb of putative super-archaic sequence from this previously unsequenced hominin, and we estimate that ∼15% of these regions have been passed on to at least one modern humans through Den→Hum introgression. Thus, our analysis suggests that at least about 4Mb of modern human genomes derives from an unknown but highly diverged archaic hominin, possibly *Homo erectus*, through at least two separate introgression events. Considering our lack of power, the true contribution could be as much as six times larger. It may be possible to identify more of this super-archaic sequence by applying ARGweaver-D to a recently sequenced set of 161 Oceanian genomes [35].

Several studies have suggested super-archaic introgression into various African populations [10, 11, 36]. However, ARGweaver-D only detected a low rate of Sup→Afr introgression, somewhat below our estimated false positive rate. Notably, however, the power to identify introgression from an unsequenced population is highly dependent on the population size of the recipient population: the larger the population, the deeper the coalescences are within that population, making it more difficult to discern which long branches might be explained by super-archaic introgression. In the case of Africans, we used a population size of 23,700, which was our best estimate from previous runs of G-PhoCS [8, 14]. If we had used a smaller population size, ARGweaver-D would have produced more Sup→Afr predictions, but possibly with a substantially higher false positive rate. In fact, one of the drawbacks of ARGweaver-D is that it depends on a demographic model, and choosing the wrong model can lead to spurious results. We have shown that ARGweaver-D is fairly robust to modest misspecifications in the model, but we recommend that careful demographic analysis be performed before running ARGweaver-D to ensure that the best possible model is used.

Finally, we detected small amounts of introgression for two additional events: Hum→Den and Sup→Nea. Because no previous evidence has been reported for these events, we expected that our predictions would be roughly in line with our false positive rates. Indeed, for the Hum→Den event, we predicted a slightly smaller fraction (0.37%) than the estimated false positive rate from simulations (0.41%). For Sup→Nea, however, we predicted 0.75% of the genome to be introgressed, which is slightly higher than the corresponding estimated false positive rate (0.65%). While these fractions are too small to draw strong conclusions, it is plausible that if *Homo erectus* mixed with the Denisovans, they may have also mixed with Neanderthals, perhaps in the Middle East; or perhaps DNA passed from *Homo erectus* to Neanderthal through the Denisovans. Altogether, given the number of gene flow events now documented among ancient hominins, it may be reasonable to assume that genetic exchange was likely whenever two groups overlapped in time and space.

## Materials and methods

### General ARGweaver-D settings

For all ARGweaver-D runs in this paper, the MCMC chain was run for 2000 iterations, with the first 500 discarded as burnin, and ARGs sampled every 20 steps thereafter. Except where otherwise noted, phase was randomized for all individuals and the phase integration feature of ARGweaver-D was used (--unphased). We used site compression throughout (--compress 10). We also used --start-mig 100, which disallows migrations for the first 100 iterations of the sampler, enabling ARGweaver-D to establish an ARG with a good general structure before exploring the migration space.

*Recombination rate*. Rather than use a recombination map calculated from modern humans, which may not be accurate for ancient hominins, we used a constant recombination rate of 5e-9/bp/generation for all analyses. This value was chosen for being somewhat between the mean and median genome-wide recombination rates (1.3e-8 and 1.7e-9 per bp per generation, respectively), and for providing reasonable power while still maintaining a low false positive rates in simulations (S1 Text). Note that all simulated data sets were nonetheless created with a real human recombination map (see “Simulations“, below).

*Mutation rate*. For real data analysis, the mutation rate map was based on primate divergence levels in 100kb sliding windows, using genome-wide alignments of human, chimp, gorilla, orangutan, and gibbon sequences (S1 Text), and scaled to an average rate of 1.45e-8/generation/site. Simulated data sets were generated by sampling rates from this map, and the same map was used for analysis.

#### Demographic model

The demographic model used in all ARGweaver-D analyses is depicted in Fig 3. The divergence times used were taken from [9], and population sizes from [8] (which were based on estimates from GPHoCS [14]). When analyzing chrX, population sizes were scaled by a factor of 0.75.

All migration events were given a prior probability *p*_*M*_ = 0.01, meaning that when a lineage encounters a potential migration event (as viewed backwards-in-time), it has a probability *p*_*M*_ of following the migrant path. In effect, this means that, when threading a new lineage into an ARG, coalescence states that include a migration are weighted by *p*_*M*_, and states that pass a migration but do not migrate are weighted by 1 − *p*_*M*_. The value of 0.01 was chosen to represent a fairly unlikely event, which gives a reasonable balance between true and false positive rates in simulations.

When analyzing non-African humans, we only included the “recent” migration bands from Neanderthals and Denisovans into humans, whereas when looking for older introgression events, we excluded the “recent” bands as well as non-African humans. Throughout this paper, all humans are placed in the same population; we do not model divergences within human populations.

Recall that ARGweaver uses a discrete-time model; 20 discrete times were chosen to span the range of relevant times, with more density near the leaves (where more coalescences occur) and to allow for coalescences between migration and population divergence events in the models. The discrete times (in kya) were: 0, 100, 200, 300, 400, 450, 500, 550, 600, 700, 950, 1200, 1450, 1700, 2000, 3000, 5000, 7000, 13,000, 15,000. Migration events occurred at half-time points including 50, 150, 250, and 350kya. Note that on this time scale, the European/African split is very recent, so that we did not model the population divergence among modern humans or recent growth in out-of-Africa populations. Similarly, we did not model the divergence between the Altai and Vindija Neanderthals, which are estimated to split only ∼ 15ky before the Altai Neanderthal individual lived. Throughout, we assume a generation time of 29 years [37].

### Efficiency of the algorithm

Without migration events, and assuming that present-day population assignment for each branch is known, the ARGweaver-D is more efficient than ARGweaver. This is because coalescence is not possible unless two branches are in the same population at the same time, so the state space of potential coalescence points will be a subset of the original state space. However, as migration events are added, coalescence points in other populations become possible, and some coalescence points may be reachable by multiple population paths (see Fig 1). Therefore, the complexity of the algorithm can quickly increase. Whereas ARGweaver’s threading algorithm had an asymptotic running time of *O*(*Lnk*^2^) (where *L* is the number of sites, *n* the number of samples, and *k* the number of time points), ARGweaver-D has a running time of *O*(*Lnk*^2^*P*^2^), where *P* is the maximum number of population paths available to any single lineage.

One way to improve the efficiency is to allow at most one migration event at any genomic location. Note that this assumption still allows multiple lineages to be introgressed at the same genomic position, if they are descended from a common migrant ancestor. This assumption is reasonable when the number of samples is small and the migration rate is low, and is set as a default in ARGweaver-D that we use throughout this paper. It has two advantageous side-effects: it avoids complex parts of the state space that could cause MCMC mixing problems (such as back-migrations, or population label switching issues). It also means that if we are modelling introgression from a “ghost” population such as a super-archaic hominin (from which we have no samples), there will be at most one (migrant) lineage in the population at any location. Therefore, the population size of ghost populations does not matter as coalescence will not occur within them.

A table giving example run times for this study is provided in S1 Text.

### Calling introgressed regions

Once ARGweaver-D has been run, introgressed tracts can be identified for each migration event by scanning the resulting ARGs for local trees whose branches follow that migration band. Throughout this paper we use a probability threshold of 0.5 to identify introgressed regions, indicating that the region was introgressed in at least half of the sampled ARGs. To predict introgressed regions for a particular individual, we compute the posterior probability that either of the individual’s two haploid lineages are introgressed. The probability of being in a heterozygous or homozygous introgressed state can be calculated as the fraction of ARG samples in which one or two lineages (respectively) from an individual are introgressed in the local tree.

The coverage of introgressed regions for an individual is computed as one-half times the coverage of heterozygous regions, plus the coverage of homozygous regions. In theory, this fails to account for sites that switch between the heterozygous and homozygous states without reaching the threshold for either, but in practice this occurs at a negligible fraction of sites.

### Analysis of hominin data

#### Data preparation

We ran a series of ARGweaver-D analyses on freely available hominin data, described in Table 2. The panTro4 chimpanzee sequence was used as a haploid outgroup. The chimp alignment to hg19 was extracted from the alignments of 99 vertebrates with human available on the UCSC Genome Browser (http://hgdownload.soe.ucsc.edu/goldenPath/hg19/multiz100way). Any region which did not have an alignment for chimp is masked in the chimp sequence.

**Table 2:** Hominin samples used in this study.

name	region	source	ID	sex	coverage	age (ky)
Vindija Neanderthal	Europe	Max Planck	Vindija33.19	F	30x	52
Altai Neanderthal	Siberia	Max Planck	Altai	F	52x	115
Denisovan	Siberia	Max Planck	Denisova	F	31x	72
Papuan	Oceania	SGDP	LP6005441-DNA_B10	F	41x	0
French Basque	Europe	SGDP	LP6005441-DNA_D02	F	36x	0
Khomani San	Africa	SGDP	LP6005677-DNA_D03	F	44x	0
Mandenka	Africa	SGDP	LP6005441-DNA_F07	F	37x	0

We downloaded samples generated by investigators at the Max Planck Institute from: http://cdna.eva.mpg.de/neandertal/Vindija/VCF; this directory contains genotype calls for several ancient genomes using a consistent pipeline and genotype caller (snpAD) throughout.

SGDP: Simons Genome Diversity Panel.

#### Filtering

For each individual, we masked genotypes with quality scores less than 20 or sequencing depths outside the range [20, 80]. For each ancient individual, we also used the filters recommended by [9] and provided here: http://cdna.eva.mpg.de/neandertal/Vindija/FilterBed. We also masked (for all individuals): any site which belongs to a non-unique 35mer, according to the UCSC Genome Browser table hg19.wgEncodeDukeMapabilityUniqueness35bp; “black-listed” sites falling under the tables hg19.wgEncodeDacMapabilityConsensusExcludable or hg19.wgEncodeDukeMapabilityRegionsExcludable; ∼ 9% of the genome for which SGDP genotype calls were not provided (85% of this set overlapped previously mentioned filters). ARGweaver-D was run in 2.2Mb windows, but we excluded any window for which any of the ancient filters, or the combined site filters, exceeds 50% of bases. In total we analyzed 1,166 autosomal windows and 52 windows on the X chromosome, covering 2.56Gb of hg19.

#### CRF calls

Introgression calls from [6] were downloaded from https://sriramlab.cass.idre.ucla.edu/public/sankararaman.curbio.2016/summaries.tgz. As recommended by the README contained therein, “set1” calls were used for Neanderthal ancestry in the Basque individual, whereas “set2”calls were used for Denisovan ancestry in both the Basque and Papuan, as well as for Neanderthal in the Papuan. For each individual, we took the set of regions with probability of introgression ≥ 0.5 in either haplotype.

#### F4 ratio

The F4 ratio statistic F4(Altai, chimp; Basque, African)/F4(Altai, chimp; Vindija, African) was calculated across the autosomal genome, and for each individual chromosome. For the African samples, we used allele frequencies across 29 African individuals from the SGDP data set (this excludes 15 individuals with the highest Neanderthal ancestry according to [13]). For this analysis we masked all sites that did not have a filter level (FL) field of 9 in the SGDP individuals. For the Neanderthals we used the same filters described previously.

### Simulated data sets (deep introgression)

We performed a series of simulations to assess ARGweaver-D’s ability to detect older migration events. Each simulated data set consists of a 2Mb region with 5 unphased diploid individuals and one haploid outgroup, mimicking the demographic histories and sampling dates of the individuals from the real data analysis. All simulations were produced with the software msprime [38].

The population tree used in the simulations is identical to the one depicted in Fig 3, and sampling dates correspond to the sample ages in Table 2. The human population size history also corresponds to the one in Fig 3. Because we were interested in levels of sharing of Hum→Nea introgressed elements, and also because the archaic populations declined sharply in size as they approached extinction, we simulated a more detailed model of population size change for the Neanderthal and Denisovan populations. We used the piecewise-constant estimates produced by PSMC [39], and published in [9]. For the Neanderthal population history, we averaged the histories produced separately for the Altai and Vindija individuals, for the time periods when they overlap. Similarly, we averaged the Denisova and Neanderthal population size estimates during the time frame of their common ancestral population (415-575 kya).

For each data set, a random 2Mb region of the autosomal genome was chosen as a template region from which we chose recombination rates and mutation rates used to generate the simulated data. We used the recombination map estimated from African-American samples [40]. For the mutation map, we used the same map as in the real data analysis (based on primate divergence levels). Missing data patterns were also taken from the template region; we applied the same ancient genome masks and mapability/blacklist masks to the simulated data. (We did not mimic the sequencing depth or quality score masks, which affected a relatively small fraction of sites).

Overall we produced several sets of simulations, each consisting of 100 2Mb regions. One set served as a control and contained no migration events. All other sets each had three types of migration (Hum→Nea at a rate of 8%, Sup→Den at 4%, and Sup→Afr at 0.5%). The rates of each event were chosen so as to have enough events per data set to be able to assess power, while still being less common than the non-migrant state. They were also chosen (by trial and error) to produce roughly similar levels of predicted introgression as observed in the real data. The simulated data sets varied in the demographic parameters used (migration time and super-archaic divergence time). A smaller set of additional simulations was produced with population sizes scaled by 0.75 to see how power might change on the X chromosome (see S4 Fig).

All false positive and true positive rates were calculated basewise; separate false positive and true positive rates were calculated for each type of migration in the ARGweaver-D model. To be classified as a true positive, the method must infer the correct type of migration in the correct individual. False positives presented here were assessed using the simulated data set with no migration.

### Simulated data sets (Nea→Hum introgression)

We also did a smaller simulation study to assess performance on the Nea→Hum event and compare performance to the CRF. Most of the settings were the same as above, except that we sampled 86 haploid African lineages and 4 Europeans, along with the two diploid Neanderthals and a haploid chimpanzee outgroup. Demographic parameters were the same as above, except that a European population diverged from the African population 100kya and had a initial size of 2100; at 42kya it experienced exponential growth at a rate of 0.002, for a present-day population size of 37236. (These parameters were roughly adapted from [22], but modified to reflect current smaller estimates of the mutation rate in humans.) We then added 2% migration from Neanderthal into Europeans at 50kya. In some supplementary analysis we also included 5% migration from human to Neanderthal 250kya.

For this analysis only, we used true haplotype phases, in order to have a fair comparison with CRF, which assumes phased samples.

#### Annotations

CDS, 3′UTR, and 5′UTR annotations were taken from the ensGene (ensembl) track on the UCSC genome browser. Enhancers and promoters were extracted from the Ensembl regulatory build dated 2018-09-25. PhastCons elements came from the phastConsElements46wayPrimates track on the UCSC Genome Browser.

### Additional methods

Further details are provided in S1 Text. All scripts used to generate simulated data, run ARGweaver-D on both simulated and real data, and analyze the results, are provided at https://github.com/CshlSiepelLab/argweaver-d-analysis.

## Supporting information

S1 Text Supplementary methods and analyses. (PDF)

S1 Table Sup→Den regions overlapping Den→Hum regions predicted by the CRF. (PDF)

S2 Table Sup→Nea regions overlapping Nea→Hum regions predicted by the CRF. (PDF)

S1 Fig False positive rates calculated from simulations, using several ARGweaver-D models. Color indicates the value of *t*_*mig*_. Shaded bars have *t*_*div*_ = 1.5Mya and solid bars have *t*_*div*_ = 1.0Mya. False positive rates are calculated base-wise using a posterior probability cut-off of 0.5. The same set of underlying data was used for all the calculations in this plot; it was simulated as in Fig 3, but with no true migration events.
(TIF)

S2 Fig Detailed simulation results. Each row shows a true migration category, and each column shows the fraction of bases predicted in the category indicated at the foot of the column. The color of each bar represents the true parameters used in simulation, as indicated in the legend, with darker colors used for the older super-archaic divergence time. Multiple bars of the same color show results on the same data set, using an ARGweaver-D model with a different *t*_*mig*_. The value of *t*_*mig*_ used by ARGweaver-D is not indicated in the plot, but increases from left-to-right: *t*_*mig*_ = 50, 150, 250, 350kya, with 50kya only shown for Sup→Afr. All the models used *t*_*div*_ = 1Mya; the plot with *t*_*div*_ = 1.5Mya is nearly identical.
(TIF)

S3 Fig Properties of introgressed regions by chromosome. The top plot shows average coverage of predicted introgressed regions per haploid genome, with darker portions representing homozygous regions. The bottom shows average length of introgressed regions by chromosome.
(TIF)

S4 Fig True positive rate for simulations on X chromosome vs autosomes. The y-axis shows true positive rates from simulations where population sizes were multiplied by 0.75 to roughly approximate X chromosome demography. Different plotting characters are used for different simulation models, as indicated in the legend. All ARGweaver-D analysis was done with *t*_*mig*_ = 250kya and *t*_*div*_ = 1.0Mya.
(TIF)

S5 Fig Frequencies of Hum→Nea introgression categories. For both the real and simulated data, Hum→Nea regions were ascertained with ARGweaver-D using a model with *t*_*mig*_ = 250kya and *t*_*div*_ = 1Mya. These regions were classified as heterozygous/homozyogus in the Altai Neanderthal (AltHet/AltHom), and in the Vindija Neanderthal (VinHet/VinHom), depending on which branches are in the migrant state in the majority of sampled ARGs. Here, the colored bars represent the fraction of Hum→Nea bases in each category for simulated data sets generated with different values of *t*_*mig*_; the error bars show 95% confidence intervals (CIs) computed using 100 bootstrap replicates across the introgressed elements. The horizontal black lines represent the amount observed in the real data, with the gray boxes showing the CIs, also obtained by the same bootstrap process. For this figure, long homozygous stretches of the archaic genomes annotated in [9] were excluded.
(TIF)

S6 Fig Frequencies of Sup→Den introgression categories. This figure is analygous to S5 Fig; here we look at putative Sup→Den regions. Because there is only one Denisovan individual, there are only two categories: heterozygous or homozygous. Note that while we expect rates of heterozygosity to decrease with migration time, the confidence intervals here are wide, as the power to detect old events is very low. As in S5 Fig, long homozygous stretches of the Denisovan genome annotated by [9] were excluded.
(TIF)

S7 Fig UCSC Genome Browser shot of a region with predicted homozygous Hum→Nea introgression in Vindija. This region on chromosome 10 has a high-probability introgressed region in both Vindija (but neither Altai) haplotypes. The top green bar indicates a predicted Hum→Nea region in Vindija, and below this is the posterior probability of introgression across the region in both Neanderthals. The variant track is similar to Fig 8. Here, we see almost identical haplotypes between Vindija and the Africans, whereas Altai shares haplotypes with the Denisovan.
(TIF)

S8 Fig UCSC Genome Browser shot of a predicted Hum→Nea region overlapping FOXP2. Exon 7, which contains human-chimp substitutions shared by Neanderthals that may be involved with human speech, is located at the very right of this plot, and is not predicted introgressed. As in Fig 7, the light green implies heterozygous Hum→Nea introgression, whereas dark green is homozygous.
(TIF)

S9 Fig Properties of Hum→Nea regions within Nea→Hum deserts. We compared the distribution of various statistics across all non-overlapping 15Mb windows in the genome (black), to the distribution within deserts of Neanderthal introgression in humans of at least 10Mb (red). We excluded any window that crosses a telomere or centromere, or where ≥ 50% of the window does not pass our filters. In the bottom-right corner of each plot is shown the Kolmogorov-Smirnov statistic p-value, indicating that there is no significant difference between the black and red distributions. The statistics shown are indicated on the x-axis label. “Hum→Nea coverage” is average fraction of the window that contains any Hum→Nea region. “Mean Hum→Nea frequency” is the average number of introgressed haploid lineages of Hum→Nea across the window (where a frequency of zero indicates no introgression, and a frequency of 4 indicates homozygous introgression in Altai and Vindija). “Mean frequency of Hum→Nea regions” is the mean frequency, among regions with Hum→Nea calls. “Altai—Vindija coverage” is difference in mean coverage between the Altai and Vindija within each window.
(TIF)
