# Supplementary material for: Mapping gene flow between ancient hominins through demography-aware inference of the ancestral recombination graph
Source: PLoS Genet. 2020 Aug 6;16(8):e1008895. doi: 10.1371/journal.pgen.1008895 (PMC7410169; doi:10.1371/journal.pgen.1008895)
Supplement: S1 Text — (PDF) [file pgen.1008895.s001.pdf]

Mapping gene flow between ancient hominins through demography-aware inference of the ancestral recombination graph: S1 Text

Melissa J. Hubisz[1,2*], Amy L. Williams[1], Adam Siepel[2*]

1 Department of Biological Statistics and Computational Biology, Cornell University, Ithaca, NY, USA

2 Simons Center for Quantitative Biology, Cold Spring Harbor Laboratory, Cold Spring Harbor, NY, USA

* mt269@cornell.edu(MJH); asiepel@cshl.edu(AS)

Supplementary Methods

Threading an ARG conditional on population structure

The main engine behind ARGweaver-D is the "threading" operation, which samples the coalescence points for a lineage that has been removed from the ARG. The threading operation uses an HMM in which the state space changes along the genome as the local tree changes. At a given genomic location, the state space is defined as the set of all possible coalescence points on the local tree, given by every time point along every branch. In the multiple population model, the state space is augmented with a third dimension indicating the "population path" of the new branch. Each population path is a vector of population assignments at every time point. This population path is sampled as part of the threading procedure, and retained in the new ARG, so that the full ARG defines the local genealogies as well as the population assignments for every lineage at each time point.

Recall that in the original ARGweaver model, time is discretized into $K+1$ points, $t_0,...,t_K$, with half-time points $t_{1/2},...,t_{K-1/2}$ between them. Coalescence and recombination events occur only at whole time points, based on their cumulative probabilities between the adjacent half-time points. In the multiple population model, both migration and population divergence are assumed to occur instantaneously at one of the discrete half-time points. This separates the coalescence process from the migration process, preventing ambiguities about the order of events in the ARG, and ensures that the number of lineages within a population is well-defined throughout a coalescence rounding interval.

Let each state be described by the vector (b,t,p), where b is a branch of the local tree where the new branch will coalesce, t is a time point of coalescence (looking backwards in time; $t=0$ is present-day), and p is a population path vector of the new branch, such that p_i gives the population of path p at time point i. The coalescence branch b is itself defined by a tuple (i,r,q) where i is the starting time of the branch, r is the parent branch, and q is the population path taken by the branch. The branch b then extends from time i to time j, where j is the time associated with parent node r. A state is only valid if $i \leq t \leq j$, and $p_t = q_t$. In other words, the coalescence must occur during the timespan of the branch, and in the same population of the branch. Coalescences may not occur between two lineages in different populations. We also assume throughout that each sample comes from a single known population (although this model could easily be extended to work for unknown or admixed samples). Therefore, when threading a leaf branch, p_0 is

known (or p_a for a leaf branch representing an ancient sample with sampling date a). As a result, if a sample comes from population P, then only states for which the population path at time zero p_0 are equal to P will be valid.

Due to these constraints, only a subset of all population paths are valid for each possible coalescent point. In the absence of migration bands, every coalescence point on the tree will be reachable by either zero or one population paths. If a coalescence point is not reachable by any population path, the point is excluded from the model, so that the number of states with non-zero probability will be reduced compared to the single population model. However, as migration bands are added, more coalescence points become reachable, and some will be reachable by multiple distinct population paths. The result is that the size of the state space - and the computational complexity of the HMM - increases as more migration bands are added, and can quickly exceed the single-population case. The cost of each migration band, in terms of run-time, depends on how many samples are in the receiving population, as well as the time of the migration band (recent migration events increase the state space more than older ones in a particular population). In order to improve both efficiency and mixing of the MCMC chain, we allow at most one migration event in any local tree in the ARG. So, ”double migration” or ”back migration” events are not allowed. When threading a branch into a local tree that already contains a migration, only states with non-migrating paths are valid. This assumption is reasonable when the rate of migration is low and the number of samples is modest.

Given this multiple population model, the threading algorithm proceeds similarly to the one described in the original ARGweaver paper [1]. The emissions probabilities (computed as the probability of the sequence data conditional on the local tree) are not affected by this model; nor are the probabilities of recombination at any point in the local tree. However, the probability of coalescence is now calculated conditional on the population path. The probability of the path also needs to be taken into account (as the product of migrating or not migrating as the branch passes through migration bands). Additionally, the symmetries exploited by ARGweaver for optimizing the forward algorithm also change. The algorithm takes advantage of the fact that the transition probabilities from state x_{i-1} to state x_i are not dependent on the branches assigned to each state, except for when the two branches are equal. Otherwise, only the coalescent times for each state matter, and the forward algorithm can be performed in $O(LnK^2)$ time, where L is the number of sites, n is the number of samples, and K the number of time points. In the demographic-aware model, the calculation also depends on the population path assigned to each state, so the complexity increaes to $O(LnK^2P^2)$ where P describes the maximum number of population paths that any lineage is able to take under the specified demographic model.

Threading migrant lineages

One additional difference is that a new type of threading has been implemented for the population model. The original model has both leaf threading (which removes and re-threads a single haploid lineage from the entire ARG), and subtree threading (which removes and resamples a series of branches, both internal and leaf). We found that neither of these algorithms are sufficient to achieve good mixing of the MCMC chain when old migration events are present, because they are not able to add or remove entire migrant haplotypes in one step.

To remedy this, we have added a branch removal algorithm that focuses on lineages and time points which may

potentially reach a migrant state. Recall that subtree threading uses a ”branch graph” structure that is designed to choose a series of removal branches from adjacent local trees, so as to minimize constraints on how these branches need to be re-threaded to maintain consistency with the remaining ARG. Given a removal branch at site i, the choice of removal branch at site $i+1$ is often deterministic, as most branches have a single analog in the neighboring tree with the exact same set of descendants. But when a branch is involved in recombination and recoalescence, then there may be two possible analogous branches to choose from. The original subtree threading algorithm made this choice randomly, and also required a Metropolis-Hastings rejection step to correct for the differing numbers of possible un-threadings in different ARGs (which is related to the number of recombination events in each ARG).

In our modified threading algorithm, we start by randomly choosing a migration band and a haploid lineage from a population which may follow this band. (For example, we may have a migration band between the 4th and 5th time interval to represent human to Neanderthal introgression, and we would randomly choose one of the Neanderthal lineages in the tree.) At the first site, we choose the branch ancestral to our chosen lineage which crosses through the time of the migration band (whether it migrates or not). We then follow the branch graph procedure, with the additional constraint that we only choose branches which span the time of the migration. In this way, there is never a random choice to make; if a branch is split in two by recoalescence of another branch onto it, then only one of the resulting segments spans the time of the migration band. In some cases, the chosen branch may be broken by recombination, and recoalesce more recently than the migration band; in this case we go back to the default of choosing the ancestral branch of the chosen lineage which crosses the migration time.

This modified-subtree algorithm guarantees that any migration event undertaken by an ancestor to the chosen lineage is completely removed from the ARG, and helps the MCMC sampler move to likely migration states, and also prevents the MCMC chain from getting “stuck” in a migration state. Note that this procedure is agnostic to whether any migration events actually exist in the ARG, and that the choice of a lineage and migration band is independent of the current sampled ARG. Therefore, the Metropolis-Hastings acceptance ratio is always 1, and the rejection step is unnecessary.

The effect of this threading algorithm is demonstrated in Fig S1-A, which shows performance with and without the new algorithm on simulated data. The algorithm appears to make very little difference in detecting the Nea→Hum migration event. We expect this is because for recent migration events, the leaf threading algorithm can effectively remove and replace entire introgressed regions. However, the algorithm improves the power for the deep introgression events significantly (except for Sup→Afr, which has very little power in either case). When the algorithm is not used, the power is much lower, though the false positive rate is still low. In this example at least, then, there is not a problem with getting “stuck” in a migration state, but in moving to the migration state.

All other analyses presented in this paper use this new threading algorithm. By default, when a migration model is being used, ARGweaver-D uses leaf threading algorithm for half the iterations, and equally divides the other iterations between the original and modified subtree pruning algorithms.

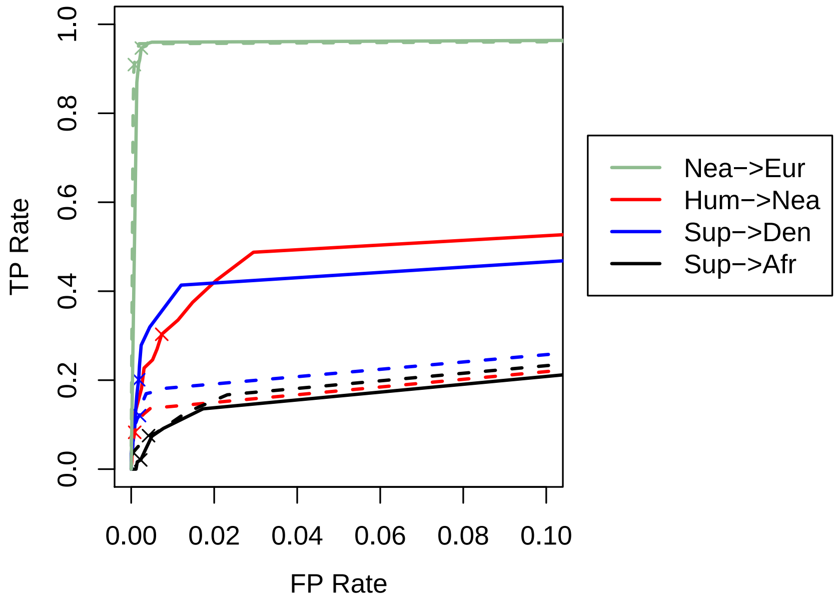

Figure S1-A: This ROC plot shows performance with (solid) and without (dashed) the modified subtree pruning algorithm. The Nea→Eur lines come from simulations in the main paper with a single migration at 50kya. The other lines come from “deep introgression” simulations with migration at 250kya and super-archaic divergence of 1Mya, and analyzed with the same model. The ”x” on each line represents the performance when a posterior probability cutoff of 0.5 is used.

Ancient sample ages

Whereas the original implementation of ARGweaver assumed that all samples came from present day, there is now an option to specify sample ages. The option `--age-file` takes a file with the ages of ancient samples, and was used throughout this study to model the Neanderthal and Denisovan lineages. All sample ages are rounded to the nearest time point in the ARGweaver-D model.

Implementing this option required a few minor modifications to the software, including removing the implicit assumption that the distance from leaf to root is the same for all leaves. An ancient sample with age t has leaves that start at time t instead of zero, and when threading this lineage, the only valid states are ones with ages $\geq t$. The code also had to be altered to ensure that lineages for an ancient branch do not contribute to coalescence probabilities in the time range $(0,t)$. Whereas the number of branches usually only decreases looking backwards in time, with ancient samples, branches may come into existence and the branch count can increase.

We did a simple simulation study to demonstrate that this option works as expected. Using `msprime` [2], we simulated 10 haploid lineages from a population of size 10000 across a 2Mb region. Four of the samples were modern day (sampled at $t = 0$); the other six were sampled at increasingly ancient times (50kya, 100kya, 150kya, 200kya, 250kya, 300kya). We used a recombination rate of 1.5e-8/recomb/bp/generation and a mutation rate of 2.5e-8/bp/generation. For this demonstration we treated samples as haploid with known phase. We then compared properties of ARGs inferred with the `--age-file` option, to ARGs inferred without the option, and also to the true known ARG. Fig S1-B

shows that this option effectively corrects for biases in the estimates caused by ignoring sample ages. It also shows that, even in this fairly extreme example with several very old samples, many statistics of the ARG are not too badly skewed even when the sampling ages are not properly taken into account.

Integrating over haplotype phase

The original ARGweaver method was designed for phased genomes; the ability to integrate over unknown haplotype phase was introduced in [3]. For reference, we include a description of phase integration here. Although many methods exist for computationally phasing genomes [4–6]; accurate phasing requires a large reference panel, which is not always available (for example, with archaic genomies). Even when computational phasing can be performed, switch errors between 0.5-5% are observed [4], depending on the reference panel size and method used. When phasing is performed prior to running ARGweaver, the ARG will be conditioned on all the phase errors. An error in the phase usually causes ARGweaver to erroneously infer recombination or mutation events. Ideally, ARGweaver would be able to create ARGs for unphased samples, or take phase uncertainty into account. To this end, we developed an approach to integrate over possible phasings while running ARGweaver. This saves the user a data processing step, eliminates the need for a reference panel, and is more robust to specific phasing errors.

When using phase integration, all individuals are randomly phased at the start of the algorithm (or if available, initialization may be done with pre-phased haplotypes). Most of the algorithm is performed conditional on the current phase of each individual. However, the leaf threading operation is performed without regard to the phase of the individual whose lineage is being re-threaded, and is followed by re-sampling the phase for that individual. The genotype phases of other individuals are held constant during this step. Leaf threading can be performed on an unphased individual by summing over the two possible phase configurations of each heterozygous site when computing the probability of the sequence data for a particular ARG. These probabilities are used as the emissions probabilities in the hidden Markov Model, which is used to sample the new threading. After the threading is complete, the phase for that individual is sampled at each heterozygous site according to the relative probabilities of the two possible phasings under the newly sampled ARG. Note that the phasings are held constant during subtree threading steps. Phase sampling is implemented in the ARGweaver source code available at `http://github.com/CSHLSiepelLab/argweaver`, using the `--sample-phase` option.

Supplementary simulation results

Out-of-Africa simulations with Hum→Nea

In addition to the out-of-Africa simulations with Nea→Eur migration presented in the main paper, we performed a second set of simulations that also included a true Hum→Nea migration event at 250kya at a rate of 0.02. We ran both CRF and ARGweaver-D on this data set. We tried two different ARGweaver-D models, one with a single Nea→Eur migration band at 50kya, and one that also included a Hum→Nea band at 250kya. The introgression

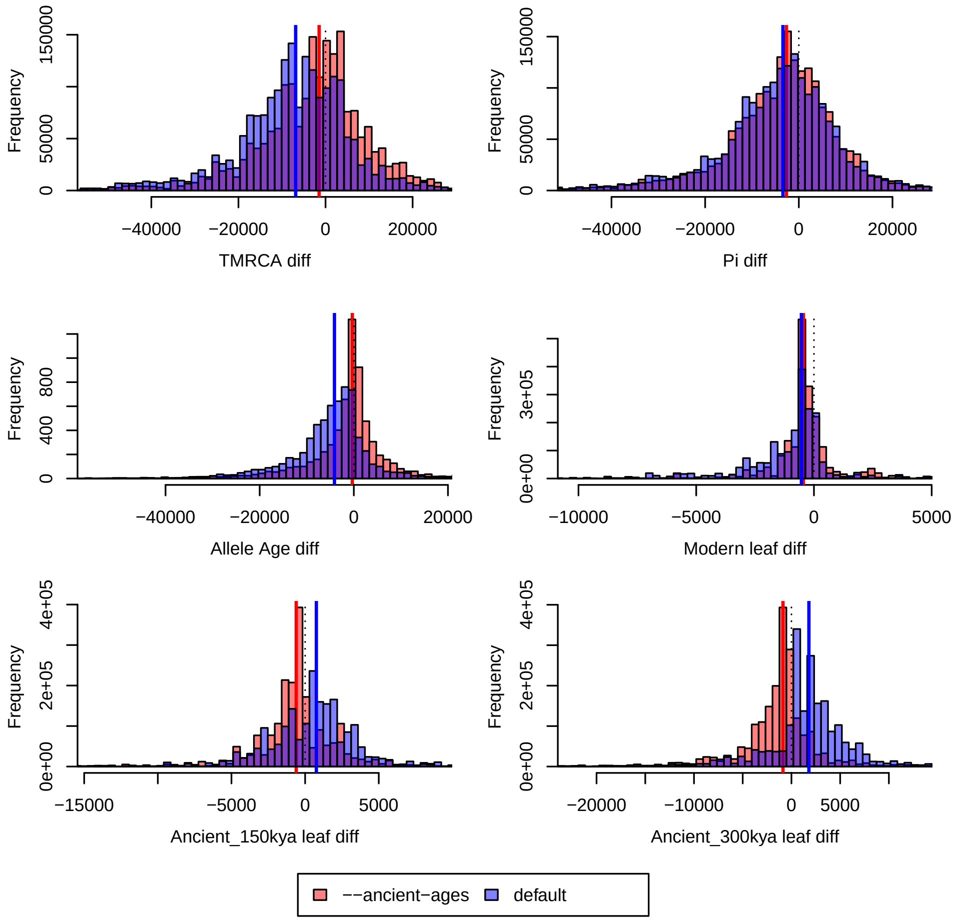

Figure S1-B: Looking at the ARG across a 2Mb region, at every base we compute the difference between the true statistic and the median from ARGs sampled across 2000 MCMC iterations. The pink distribution shows the ARGs inferred while accounting for ancient sampling dates; the blue uses the default parameter. (Purple is the overlap between the two). The dotted black line is at $x = 0$, and the red and blue lines are at the medians of the pink and blue distributions. The statistic for each plot is named in the x-axis, and the names are as follows: TMRCA (time to most recent common ancestor, in generations); Pi (average distance between two leaf nodes, in generations); Allele age (age of derived alleles); Modern leaf (coalescence time of a leaf node for a present-day sample); Ancient_150kya leaf (coalescence time of a leaf sampled 150kya), and Ancient_300kya leaf (coalescence time of a leaf sampled 300kya).

predictions are summarized in Fig S1-C. When ARGweaver-D has only one band, it performs similarly to CRF, though with a somewhat lower false positive rate. Both methods mis-classify a small fraction of Hum→Nea regions as Nea→Eur (9.0% for CRF and 7.6% for ARGweaver-D). When a second band is added to the ARGweaver-D model, the true positive rate (34%) for correctly identifying Hum→Nea is identical to what we saw in the "deep introgression" simulations presented in the main paper. However, a large fraction (38%) of the Nea→Eur regions are incorrectly classified as Nea→Hum. We are not sure why the misspecification is so much higher in one direction than the other. However, we have observed in general that getting the correct directionality when there are migrations between sister populations is difficult. This is one reason for excluding non-African populations when looking for older migration events.

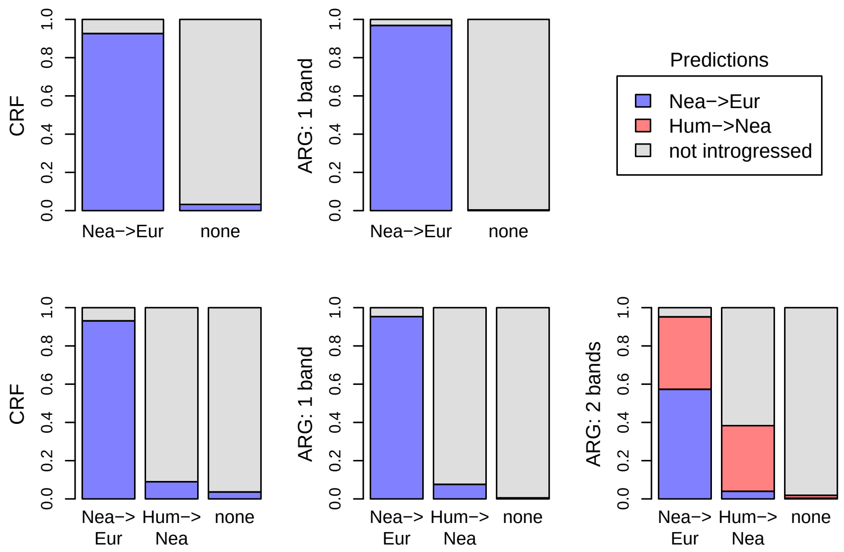

Figure S1-C: On the top is shown predictions for a set of simulations with only Nea→Hum introgression. Each bar represents a true (known) category; the colors show predictions for this category using the CRF (left) and ARGweaver-D with a single migration band (right). On the bottom are results where there are two true migration events. Here there are two sets of ARGweaver-D results; one with only the Nea→Hum band, and one that also has a Hum→Nea band.

Recombination rate analysis

Most of the analysis in this paper was done assuming a constant recombination rate across the genome. While this is unrealistic, little was known about the recombination map in Neanderthals and Denisovans at the time this work was done (though see [7]). Furthermore, othing is known about possible super-archaic recombination maps.

To explore the effects of recombination rate misspecification, we compared the performance on our "deep introgression" simulations with four different recombination settings: one with the true map used in the simulations, one with a constant rate of 5e-9/recomb/generation/base-pair, another with a higher rate of 1e-8/recomb/generation/base-pair, and one with an incorrect recombination map. All simulations were created with a true recombination map sampled from some a random region of the human recombination map generated from African-American samples [8];

the incorrect maps were sampled from a different random region of the human genome. The results are shown in Fig S1-D.

Overall, the recombination rate seems to have the biggest impact on the false positive rate. Except for in Sup→Afr, where the power is low everywhere, the best performance is when the true recombination rate is used, and the worst are when a too-high or wrong rate is used. Using a constant rate of 5e-9 gave intermediate performance, and fewer false positive than other incorrect maps. Because hotspots identified in human may not apply to Neanderthal, Denisova, or super-archaic hominins, we chose to use the low recombination rate.

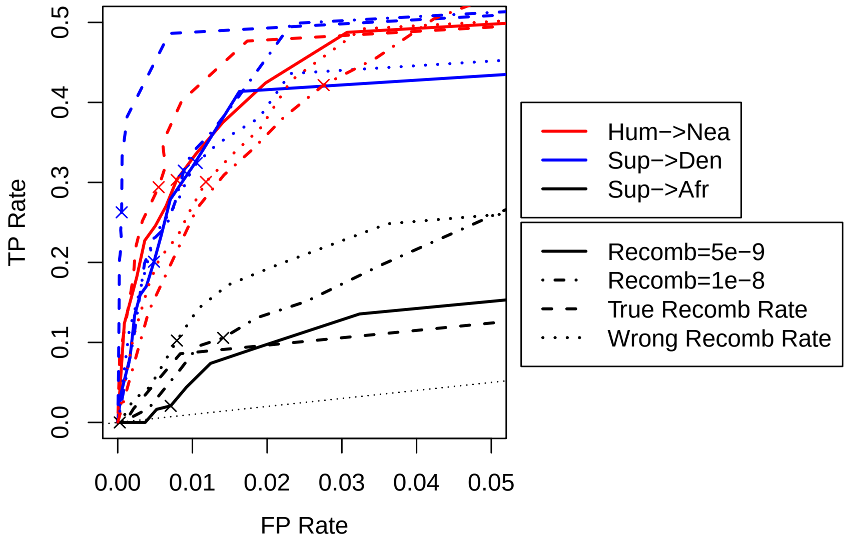

Figure S1-D: Performance on the “deep introgresion” simulations, in which the only difference between ARGweaver-D runs is the recombination map used for analysis.

Population size analysis

In the main paper, we did not investigate the sensitivity of our method to mis-specified population size. This is because, unlike the time of migration or super-archaic divergence, there are established population genetic methods for estimating population size histories, even for small samples (i.e., [9–12]). However, as mentioned in the discussion, using the wrong population size in the ARGweaver-D model may affect the performance of the method, and in some cases may cause an increase in false positives. In this section we explore the extent of this effect.

We used the same “deep introgression” simulations as presented in the main paper, focusing on the set with $t_{mig} = 250$kya and $t_{div} = 1$Mya. We then used ARGweaver-D to call introgressed regions, using the ARGweaver-D model presented in Fig 3, with correct values of t_{mig} and t_{div}, but perturbing the population sizes in the model. For each population in turn, we multiplied the population size by a factor f. We did this analysis for each population— Human, Neanderthal, Denisovan, Super-Archaic, and ”all” (i.e., the entire population tree, including ancestral populations— and for each of the values $f = (0.25, 0.5, 0.9, 1.0, 1.1, 2.0, 4.0)$. The results are presented in Fig S1-E (which shows the effect on true positive rates) and Fig S1-F (which shows the effect on false positive rates).

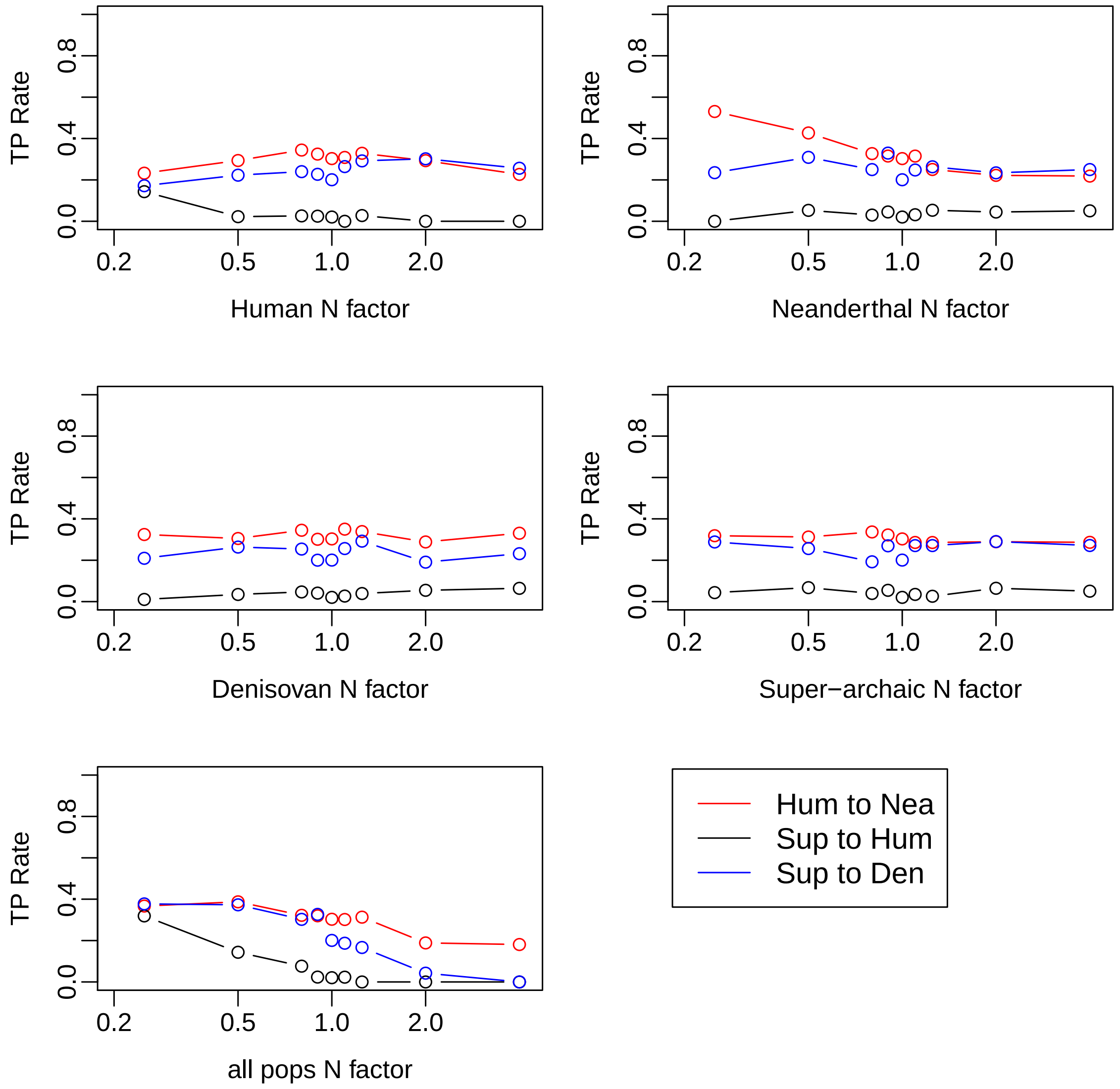

Figure S1-E: True positive rates for calling introgression, for migration events indicated by the line color, when varying the population size used in the ARGweaver-model by factors indicated on the x-axis. In the bottom left plot, all population sizes are multiplied by the factor in the x-axis.

Overall, the power of the method does not seem to be strongly affected by the population size in the model, although in a few cases, power increases when introgression is being detected into a population for which the true population size is much bigger than the one used in the model.

We see a similar pattern with the false positive rates; there is virtually no effect when the population sizes in the model are too large, but when they are too small for some population, the false positive rate for introgression events into that population can increase. This is most prominent for the Sup→Afr event, where the false positive rate gets as high as 0.07 when the population size is underestimated by a factor of 0.25, and about 0.025 when it is underestimated by a factor of 0.5. For the Hum→Nea event, the false positive rate gets as high 5% when the Neanderthal population

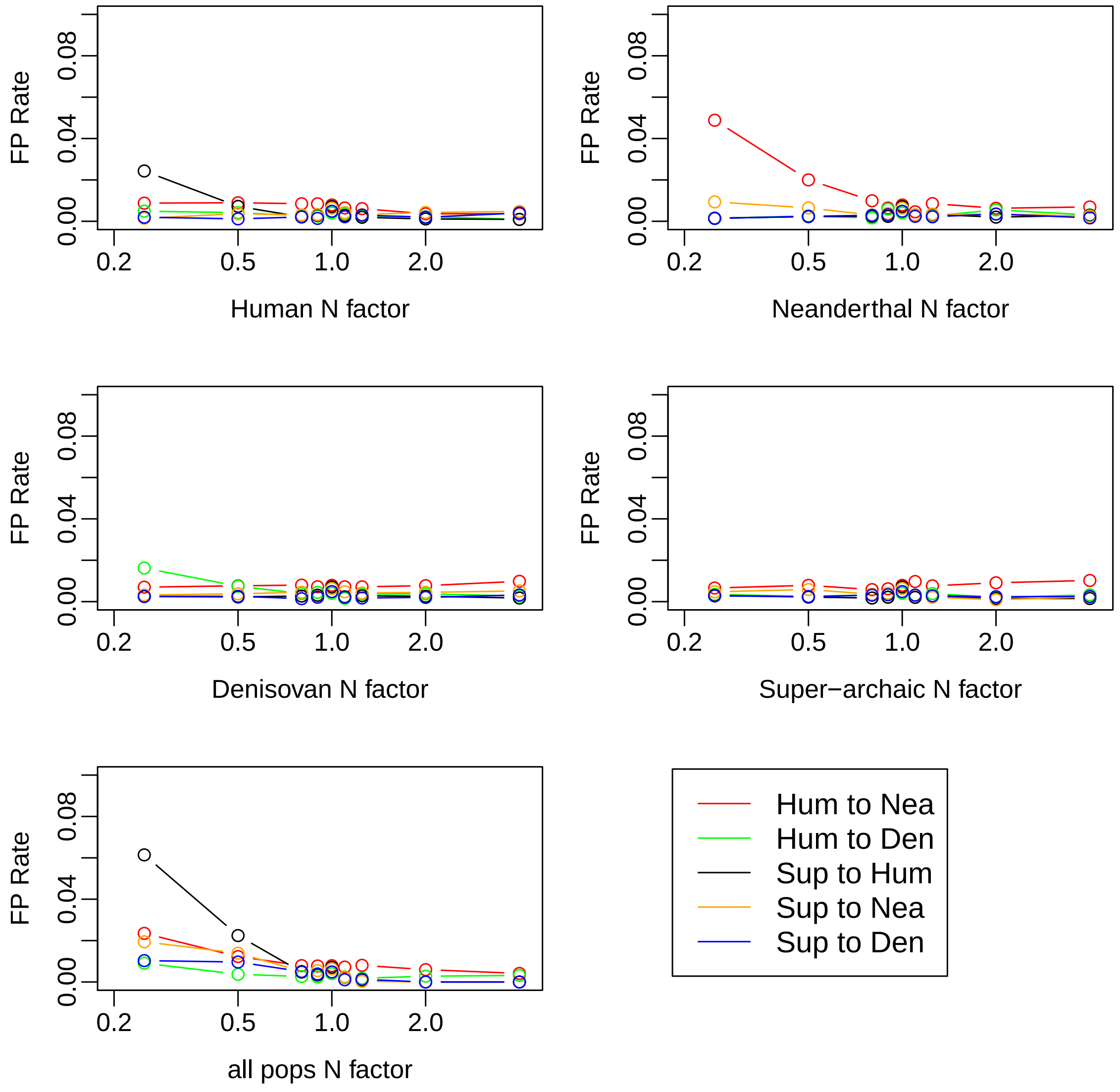

Figure S1-F: As in Fig S1-E, but showing false positive rates. This includes some migration events (Hum→Denand Sup→Nea) which were not simulated, so for which we can only compute false positive rates and not true positive rates.

size is multiplied by 0.25, or 2% when it is multiplied by 0.5. It is curious that we do not see the same effect for the Sup→Den or Sup→Nea, and only a very muted similar effect for Hum→Den. We believe that the effect will be more pronounced when there are more samples in the population (because there are more coalescence events to model). It also seems that the presence of a sister population for Neanderthal and Denisovans, as well as their overall small population size, makes false positives for the Sup→Nea and Sup→Den events less likely than for the Sup→Afr.

It is also worth noting that, as expected, there is no significant effect when the super-archaic population size is misspecified. Because we restrict the model to a single migrant lineage at any local tree, there will never be more than one lineage in the super-archaic population, and therefore coalescence events will not occur in that population, and its

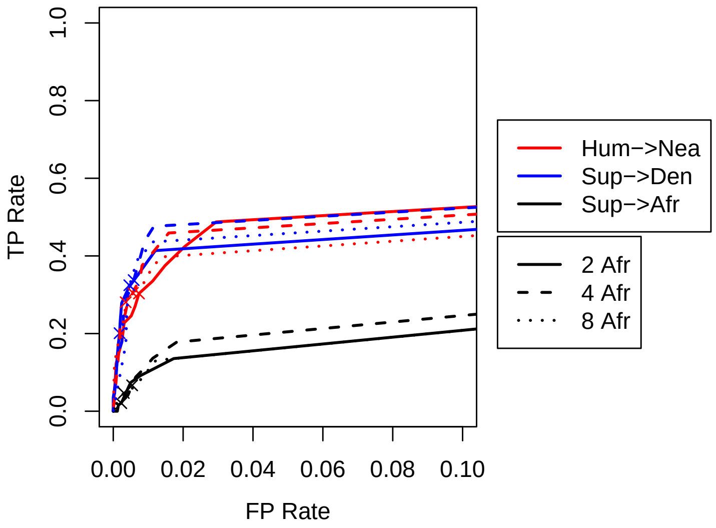

Figure S1-G: Effect of using more African individuals in the analysis. Here, we used the same "deep introgression" simulations as in the main paper, but sampled and used more African individuals in the ARGweaver-D analysis. Both the simulated data and the ARGweaver-D model had a migration band at 250kya and super-archaic divergence of 1Mya. The "x" on each line represents the performance when a posterior probability cutoff of 0.5 is used.

size is irrelevant to the ARGweaver-D model.

In summary, there is some possibility of increased false positive rates when the population sizes are misspecified (and in particular under-specified in the ARGweaver-D model), but for the models used in this paper, the misspecification has to be quite serious in order to produce a large effect. The model seems robust to over-estimation of population size, or under-estimation of at least roughly 20%. Using the best population size estimates available for the relevant data should keep false positive rates under control.

Number of African samples

In theory, larger numbers of African samples might be expected to improve performance for finding Hum→Nea introgression events, as the introgressing ancient human population is most closely related to modern Africans, and seems to be equally related to various diverged African populations [3]. It is also possible that more African samples could boost the power of detecting Sup→Den regions, as they contribute more information about the ancestral archaic population. However, in practice we observed that power using 2 Africans was similar to using 4 or even 8 African individuals (Fig S1-G). We are not sure why this is, but suspect that the MCMC sampler does not mix as well as more individuals are added. Because ARGweaver-D is faster with fewer individuals and it does not seem to have much effect on performance, we did our main analysis with two Africans.

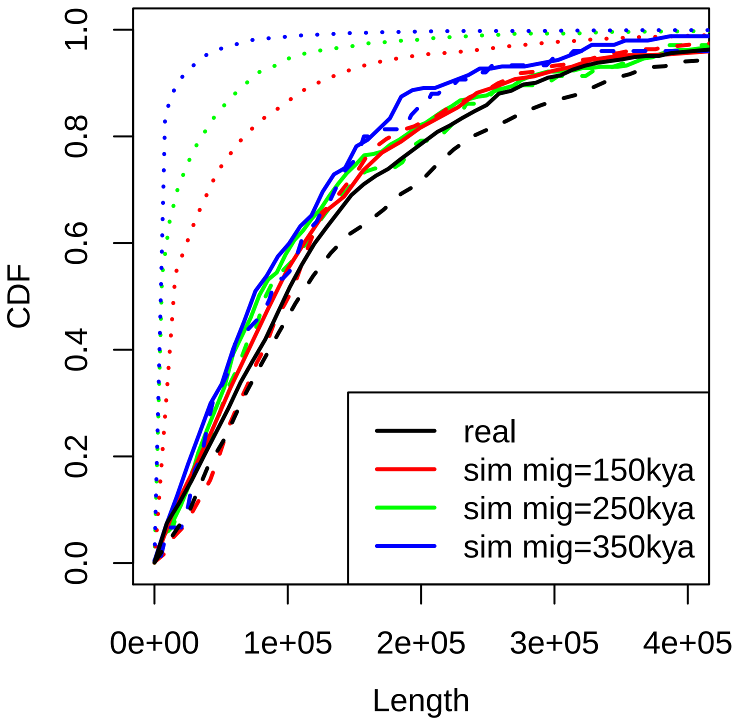

Figure S1-H: The distribution of lengths in real vs simulated Hum→Nea regions. The dotted lines show the true distribution of lengths in three simulated data sets, each produced with different migration times, indicated by the color. The solid lines show the distribution of regions found by ARGweaver-D in the simulated and real (black) data sets when analyzed with a model with a migration band at 250kya. The dashed lines show the same except with a migration band at 150kya.

Supplementary analysis

Lengths of real vs simulated introgressed regions

The lengths of introgressed regions should be informative for the time of migration. However, there is more power to detect longer regions, creating a strong ascertainment bias that makes interpretation of the lengths difficult. The rate of recombination is also an important factor affecting the distribution of lengths, and the recombination rates in Neanderthal and Denisovan are not well characterized. Still, we looked at the distribution of lengths in our predicted set, and compared to both the true and predicted regions in simulations with different migration times.

Overall, our predicted elements were somewhat longer (median 105kb) than those observed in our simulations (median lengths ranging from 71kb-89kb), but the distributions were largely overlapping (Fig S1-H). We concluded that the distribution of lengths is not a useful summary statistic for inferring model parameters.

We would also expect that the Hum→Nea segments would be longer in the Altai than the Vindija Neanderthal, since the Altai Neanderthal is a much older sample. We explored this statistic in our simulations; the distributions are shown in Fig S1-I. Again, we find that ascertainment bias makes it difficult to assess. When $t_{mig} = 150$kya, we see that Altai introgressed regions are longer than in Vindija, but after ascertainment, the distributions are very close. When

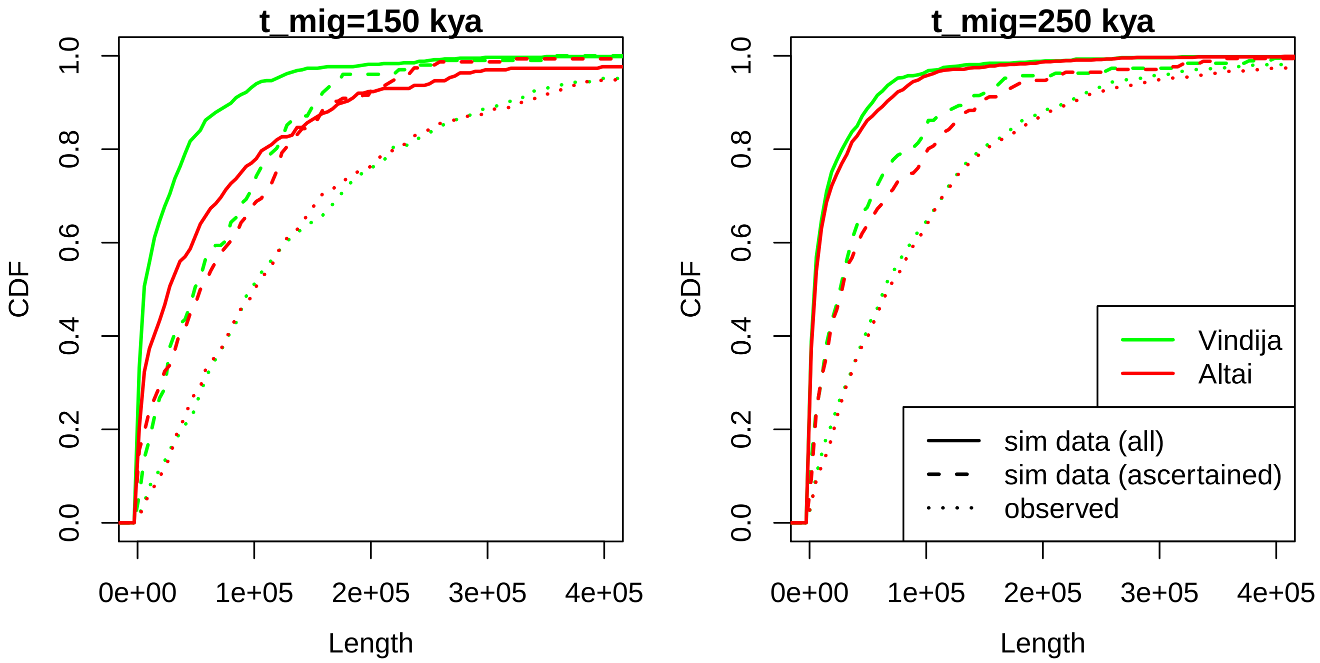

Figure S1-I: The distribution of Hum→Nea introgressed segment lengths in the Altai (red) and Vindija (green), in simulations and the real data.

$t_{mig} = 250$kya, there is not a large difference in even the distributions before ascertainment. In the real data, we also see very little difference in the length distribution of Hum→Nea segments between Altai and Vindija.

Validation of super-archaic regions in SGDP individuals

We further explored the 27Mb of the genome which was putatively identified as Sup→Den. This category had the strongest prior evidence for super-archaic introgression [13, 14], and is the only super-archaic category for which the amount detected by ARGweaver-D (1%) significantly exceeds the false positive rate estimated from simulations (0.5%). We first identified variants in Sup→Den regions that map to the migrant lineage in our data set (which included the Denisovan, two Neanderthals, SGDP individuals Khomani_San_1 and Mandenka_2, and chimpanzee); there are 15,470 variants over 16.8Mb of unmasked Denisovan sequence. This suggests an average substitution rate is 9.2e-4/bp, which translates to a branch length of 1.8My (using a mutation rate of 1.45e-8/bp/generation and a generation time of 29 years). However, we expect that this estimate is biased upwards, as Sup→Den regions with more variants are easier to detect. We compiled a VCF file containing all the substitutions on the super-archaic haplotype.

We next looked at all 279 individuals in the SGDP data set, comparing their divergence to Neanderthals and Denisovans in each region. If the Sup→Den prediction is correct, then the Denisovan divergence should be high for all humans, and not just the two humans used in the ARGweaver-D analysis. Two example regions are shown in Fig S1-J. As described in the caption, and explored further below, most Sup→Den regions do indeed show higher divergence to the Denisovan across the SGDP individuals than to the Neanderthal, excepting individuals with Den→Hum introgression in that region. There are a small number of Sup→Den regions, such as the one shown in Fig S1-JB,

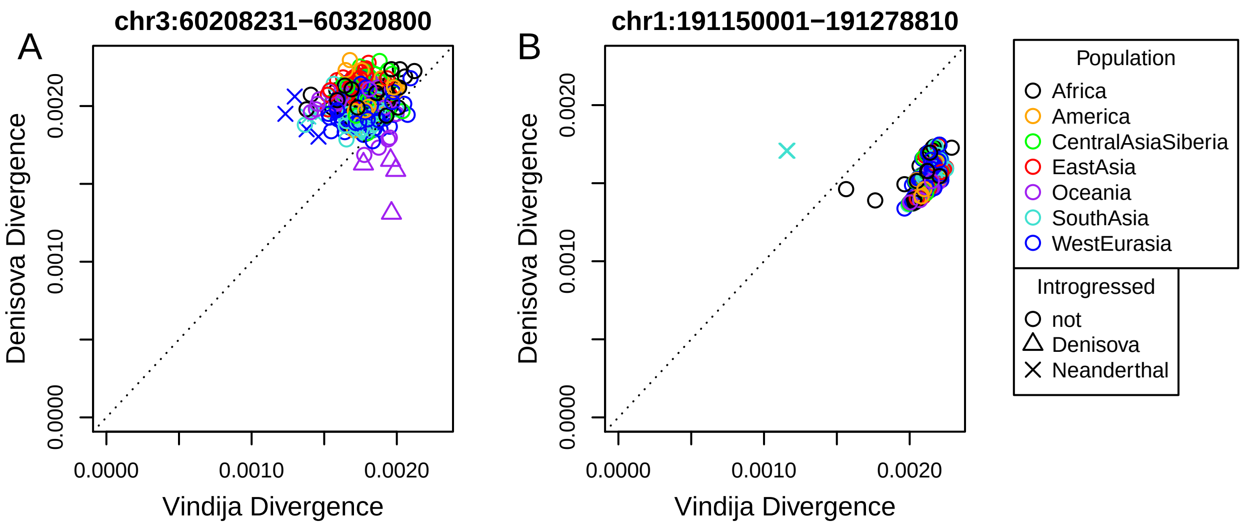

Figure S1-J: **Average divergence of SGDP individuals to Neanderthal and Denisovans in example Sup→Den regions** The color represents the population of each sample, whereas the symbol indicates whether introgression has been detected in this individual in at least half of the plotted region (according to the CRF method). A) This region shows the expected pattern for Sup→Den: most individuals have higher divergence to the Denisovan than to the Vindija Neanderthal. However, a few Oceanian individuals who have Denisovan introgression in this region have lower Denisovan divergence. Similarly, some European individuals with Neanderthal introgression also show a decreased Neanderthal divergence. B) This is a less typical Sup→Den region that is likely a false positive, as most SGDP individuals show lower divergence to the Denisovan than to Neanderthal. It is interesting that one of the outlying African dots represents an individual used in the ARGweaver-D analysis (Khomani_San_1).

where the Denisovan divergence does not exceed Vindija divergence in most humans and might be false positives of the ARGweaver-D approach.

While looking at example plots is helpful, we want to summarize the properties across all Sup→Den regions. We define a statistic f, which is the fraction of SGDP individuals in a given region for which the Denisovan divergence is greater than the divergence to either Neanderthal. This statistic can be visualized as the fraction of individuals that fall above the diagonal in plots such as those in Fig S1-J. In each region we exclude any individuals with Neanderthal or Denisovan introgression (as assigned by the CRF [15]). We computed f for each of the 161 putative Sup→Den regions with length $\geq$ 50kb, as well as for 262 putatitve Sup→Nea regions, 384 putatitve Sup→Afr regions, and 500 100kb regions randomly selected from regions of the genome without any ARGweaver-D introgression assignment. The distributions of f for each set of regions are shown in Fig S1-K. We see that for about 80% of the Sup→Den regions, f is close to 1. There are 29 Sup→Den regions with $f < 0.9$ (including the region shown in Fig S1-JB), and which might be best regarded as false positives.

For the Sup→Nea regions, where we would expect most individuals to have higher Neanderthal than Denisovan divergence, we see a similar large shift towards small f; most SGDP individuals are closer to Denisovans than Neanderthals in these regions. In this case, 73% of the regions have at least 90% of individuals closer to the Denisovan.

It is important to note that while this analysis provides a check on ARGweaver-D's predictions, and identifies

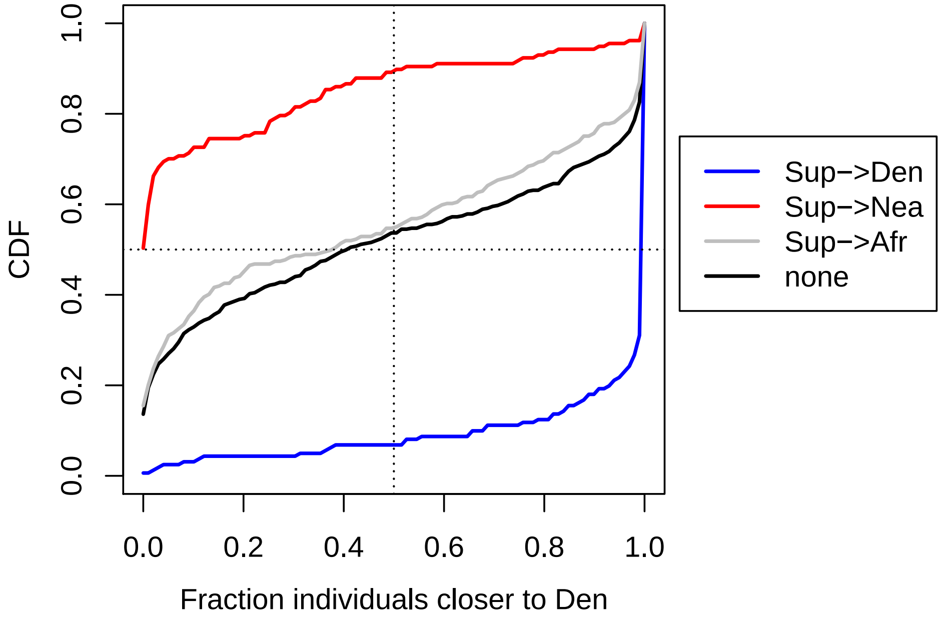

Figure S1-K: **Distribution of the fraction of individuals with higher Denisovan vs Neanderthal divergence**. Each cumulative distribution shown here is taken across a set of regions assigned to a particular introgression category by ARGweaver-D. For each region, we calculate the number of individuals for which the Denisovan divergence is higher than the Neanderthal divergence (excluding individuals with calls of Neanderthal or Denisovan introgression by the CRF). We see that Sup→Den regions have a high proportion of individuals more closely related to Neanderthal, and the opposite pattern in Sup→Nea regions. Both putative Sup→Afr and non-introgressed regions are very slightly biased towards Neanderthal ancestry.

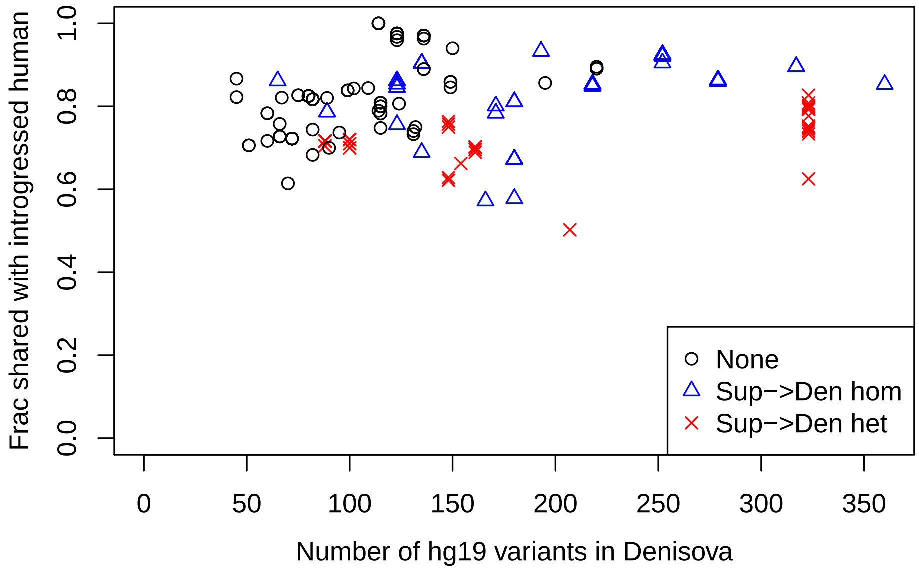

Figure S1-L: **Fraction of shared variants with Denisovan, for individuals with Denisovan introgression.** Each point is calculated for a particular genomic region and individual with Denisovan introgression in that region. The x-axis shows the number of Denisovan hg19 differences; the y-axis shows the fraction of these variants shared with the individual. The colors represent the type of region; blue regions are homozygous Sup→Den regions, red regions are heterozygous Sup→Den regions, and black are regions without any ARGweaver-D introgression calls in Africans or archaics.

some potential false positives, it does not imply that the remaining regions are true positives. Other scenarios, such as balancing selection, could also produce long regions of high divergence that may be virtually indistinguishable from super-archaic introgression. But this analysis does show that the signal identified using only two humans usually holds across a much larger set.

Analysis of Sup→Den regions passed to modern humans

Presumably, if there is super-archaic introgression into Denisovans, and later Denisovan introgression into Oceanian and Asian humans, then it seems likely that these modern humans harbor super-archaic alleles passed through the Denisovans. Indeed, 15% of our Sup→Den regions overlap regions with Den→Hum introgression calls in the SGDP (24 out of 161 regions, excluding regions with lengths <50kb). We looked into this by comparing the variants on the super-archaic lineage with those observed in individuals with Denisovan introgression (according to the CRF calls). Fig S1-L shows the fraction of shared Denisovan variants vs. the number of hg19/Denisovan variants for individuals that are annotated with Hum→Den introgression by the CRF method.

The black points in Fig S1-L show the fraction of shared variants in regions without any ARGweaver-D introgression calls in Africans or archaics. We see that the fraction of shared alleles is high (between 60-100%) for these regions, though the overall number of variants is moderate. The blue points show the same values in regions that have

been identified as Sup→Den introgressed in both Denisovan lineages. For the most part, we also see high fraction of shared variants, although the absolute number of variants is much higher overall. This suggests that the individual is sharing super-archaic alleles, as the majority of these variants occur on the super-archaic branch. Finally, the red points show a subset of Sup→Den where the super-archaic introgression is only found in one of the Denisovan lineages, so that our sampled Denisovan has both a "super-archaic" and a "Denisovan" haplotype. The red points with the lowest fraction shared may represent individuals who received the Denisovan haplotype.

One consideration here is that there is likely a bias towards identifying Denisovan introgression in humans when the Denisovan and human both share the super-archaic haplotype, because introgression will be very easy to detect with such a large number of shared variants. This may explain why there are not many red points with lower fractions of shared Denisovan variants in Fig S1-L. Regardless, this analysis shows that many individuals with Denisovan introgression share alleles that are predicted introgressed into Denisovan from a super-archaic hominin.

Analysis of Sup→Nea regions passed to modern humans (and the hg19 reference sequence)

We did a similar analysis on regions identfied as Sup→Nea, this time looking at the overlap between these regions and Nea→Hum regions in SGDP humans. 35% of our Sup→Nea regions overlap regions with Nea→Hum introgression according to the CRF predictions (55 out of 157 regions, excluding regions with length <50kb). Fig S1-M summarizes these regions; there are many more points in this plot than in Fig S1-L because there are many more SGDP samples with Neanderthal introgression.

The first surprising aspect of these results is that there was one region (chr6:8450001-8563749) classified by ARGweaver-D as Sup→Nea, but which had only 13 hg19 differences across 79kb of unmasked Neanderthal sequence (giving an hg19/Neanderthal divergence of only 0.016%). After closer inspection, we suspect that this region has Neanderthal introgression in the hg19 reference sequence. Among SGDP individuals with annotated Neanderthal introgression in this region, there are between 1 and 18 homozygous hg19 differences. Among the other SGDP individuals, there are between 68 and 369 homozygous hg19 differences. (This excepts one individual, Saharawi_1, fosr whom CRF introgression calls were not made and which has only one hg19 difference; we presume it is also Neanderthal introgressed. The Saharawi are a Northern African population that has been found to have almost as much Neanderthal introgression as non-African populations [14]). Given that the reference sequence is largely African-American, we should expect that it would contain some Neanderthal ancestry. While the theory of possible introgression from super-archaic introgression into Neanderthal does not yet have strong support, if the annotated Sup→Nea regions are correct, this would be an example of archaic hominin ancestry in the hg19 reference sequence, passed through Neanderthals. We also note that we found one other region (chr10:88106371-88206370), from our set of 500 randomly selected 100kb regions without ARGweaver-D introgression calls, in which the number of hg19 differences is < 4 for SGDP individuals with Neanderthal introgression, and much higher (median: 75) for other SGDP individuals. As our analysis in this section only spanned 2.4% of the genome, a genome scan for Neanderthal introgression on hg19 would discover many more such regions.

Beyond the observation of Neanderthal (and possibly super-archaic) ancestry in hg19, Fig S1-M shows that there

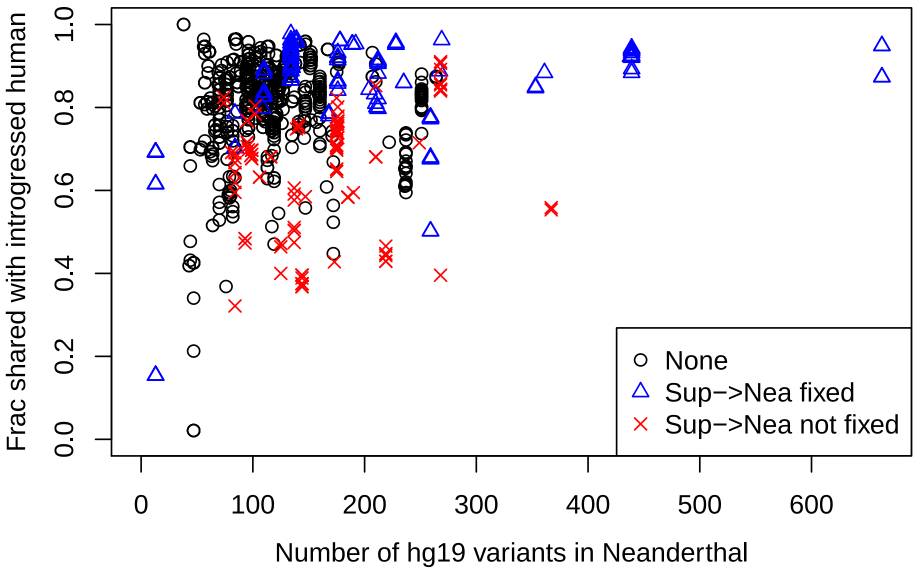

Figure S1-M: **Fraction of shared variants with Neanderthal, for individuals with Neanderthal introgression.** Each point is calculated for a particular genomic region and individual with Neanderthal introgression in that region. The x-axis shows the number of Neanderthal hg19 differences in the region; the y-axis shows the fraction of these variants shared with the individual. The colors represent the type of region; blue regions are Sup→Nea regions, red regions are a subset of Sup→Nea that are not fixed in our Neanderthal sample, and black are regions without any ARGweaver-D introgression calls in Africans or archaics.

are indeed a number of regions annotated as Sup→Nea with a large number of hg19 variants, which are also shared to a high degree with Neanderthal-introgressed humans in the SGDP. Again, we see that the red points (representing non-fixed Sup→Nea regions) sometimes share fewer variants with Neanderthal, suggesting that these individuals are introgressed with the 'Neanderthal' haplotype rather than the 'super-archaic' haplotype.

Overall, the analysis of both Sup→Den and Sup→Nea regions show that these regions have a high number of variants compared to non-annotated regions, and that these variants are often shared with humans with introgression from Denisovans or Neanderthals (respectively). While the Sup→Nea event is not well supported, and the Sup→Nea regions may simply be highly diverged Neanderthal regions, there is stronger support for the Sup→Den migration, and it seems that humans with Denisovan ancestry must also harbor some variants from more diverged hominin species as well.

Functional enrichment analysis of introgressed regions

We checked for enrichments or depletions of various functional elements in our introgressed segments. However, the interpretation of these numbers is not straightforward, as the power to detect the segments is confounded by many factors, such as local population size, mutation rate, recombination rate, and sequence quality filters. Indeed, even for introgression into humans where the detection power is quite high, these depletions have been difficult to interpret (see Discussion). The enrichments are shown in Fig S1-N. It is clear that biases for functional elements depend on the type of introgression event (from a sample population or super-archaic). We found a 1.15x enrichment of ensembl CDS regions in our Hum→Nea calls, which is most likely explained by higher power with lower effective population size. Perhaps the most interesting aspect is that the enrichment Hum→Nea in most functional categories (including CDS, phastCons, promoters) is larger in the Vindija Neanderthal than the Altai, despite the fact that the Vindija and Altai Hum→Nea regions are highly overlapping (55.6% of the combined set are called in both individuals). Again, it appears that functional elements were not lost in the duration between the Altai and Vindija individual's lifetimes, suggesting an absense of negative selection acting against these regions.

Deep introgression analysis with other models

In the main paper, we presented results using an ARGweaver-D model in which all migration times (t_{mig}) were set to 250kya and super-archaic divergence time (t_{div}) to 1Mya. We also ran ARGweaver-D genome-wide with t_{mig} =150kya and t_{div}=1.5kya. The coverage of the resulting elements is shown in Fig S1-O.

The results using this model are qualitatively similar to those presented in the main paper; the largest coverage is in Hum→Nea, with increased coverage on the X chromosome, and a somewhat smaller depletion from Altai to Vindija on the X. We again see similar low levels of all other introgression categories, and the same depletion for Sup→Den on the X chromosome. The coverages of predicted introgressed Hum→Nea and Sup→Den elements are about half what is presented in the main paper. This is consistent with our simulation results which show that power is much lower using this model when the true model more closely matches the one used in the paper, and further supports our

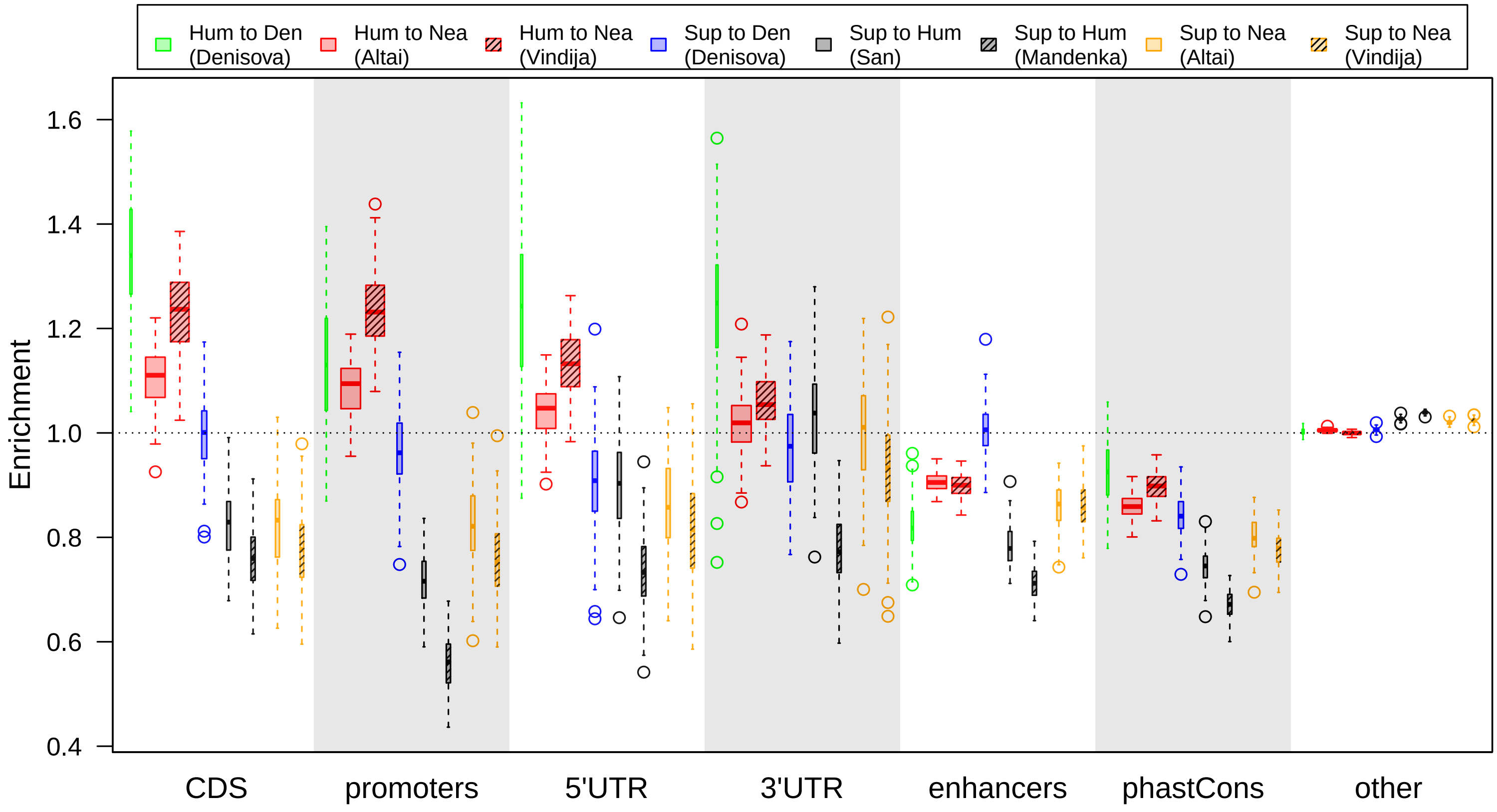

Figure S1-N: **Enrichment of predicted introgressed regions within different annotation groups.** Enrichment is calculated as the number of introgressed bases called within a particular category, divided by the number expected assuming that the introgression calls are independent of the annotations. The distributions shown in the box plots are calculated from 100 bootstrap replicates over the introgression calls. The width of each bar is proportional to the total coverage of the predicted introgressed elements genome-wide. These enrichments are likely highly influenced by factors affecting the power and false positive rates of the calling algorithm.

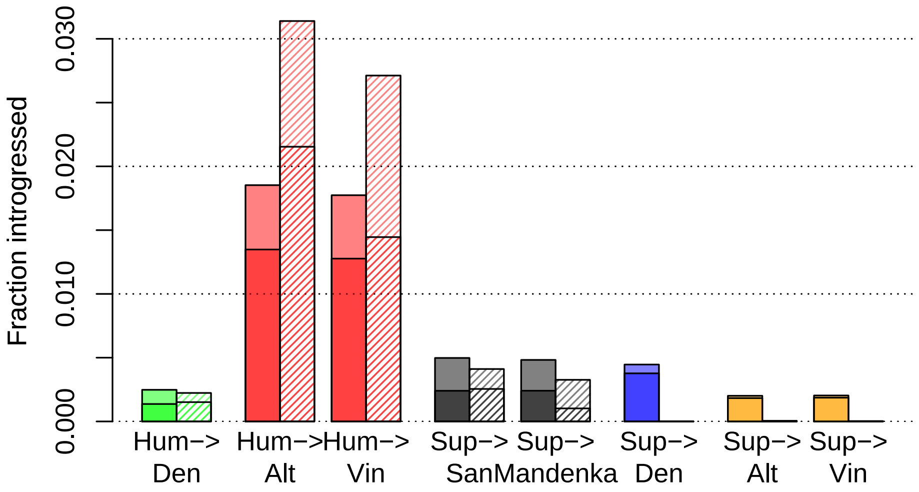

Figure S1-O: **Introgression coverages under alternative demographic model**. Genome-wide coverages using a migration time of 150kya and a super-archaic divergence of 1.5Mya. Solid bars show autosomal coverage; striped bars show X-chromosome coverage. The dark bottom halves of each bar represent regions that were also predicted by the model used in the main paper.

claims that the Hum→Nea migration was quite old, and that the super-archaic divergence was low. We repeated the analysis of the frequencies of Hum→Nea regions using results analyzed with $t_{mig} = 150$kya; the results are shown in Fig S1-P. Due to lower numbers of regions predicted, the results are noisier, but do not change the overall conclusions from the analysis with $t_{mig} = 250$kya.

We also did a genome-wide run in which we included the Papuan and Basque, along with the two Africans and archaic individuals. We analyzed the data as before, using $t_{mig} = 250$kya and $t_{div} = 1$Mya, but also added migration bands from Nea→Hum and Den→Hum at 50kya, for a total of 7 migration bands. The introgression coverages for this run are presented in Fig S1-Q. Although there are moderate differences in the absolute level of introgression predicted, the results agree well with those presented in the main paper. We do see an increase in the amount of Hum→Den and Hum→Nea regions, which most likely can be explained by the algorithm calling the incorrect direction of migration for some instances of Den→Hum and Nea→Hum.

Fraction of Neanderthal genome introgressed from humans

Using the true and false positive rates in from the deep introgression simulations in the main paper, we can make a rough estimate of the total amount of Neanderthal genome introgressed from ancient humans. If the fraction of the genome predicted to be introgressed is x , then the total amount is predicted to be $(x - FP)/TP$. These numbers are of course very rough because there are many unknown demographic parameters in the model. Using the results in the main paper, this would predict that 7.4% of the Altai autosomal genome is introgressed from Neanderthal, and 7.2% of the Vindija. If we instead use the simulation and real results from the previous supplemental section, where the demographic model has a migration at 150kya, we get predictions of 10.8% for Altai and 10.3% for Vindija.

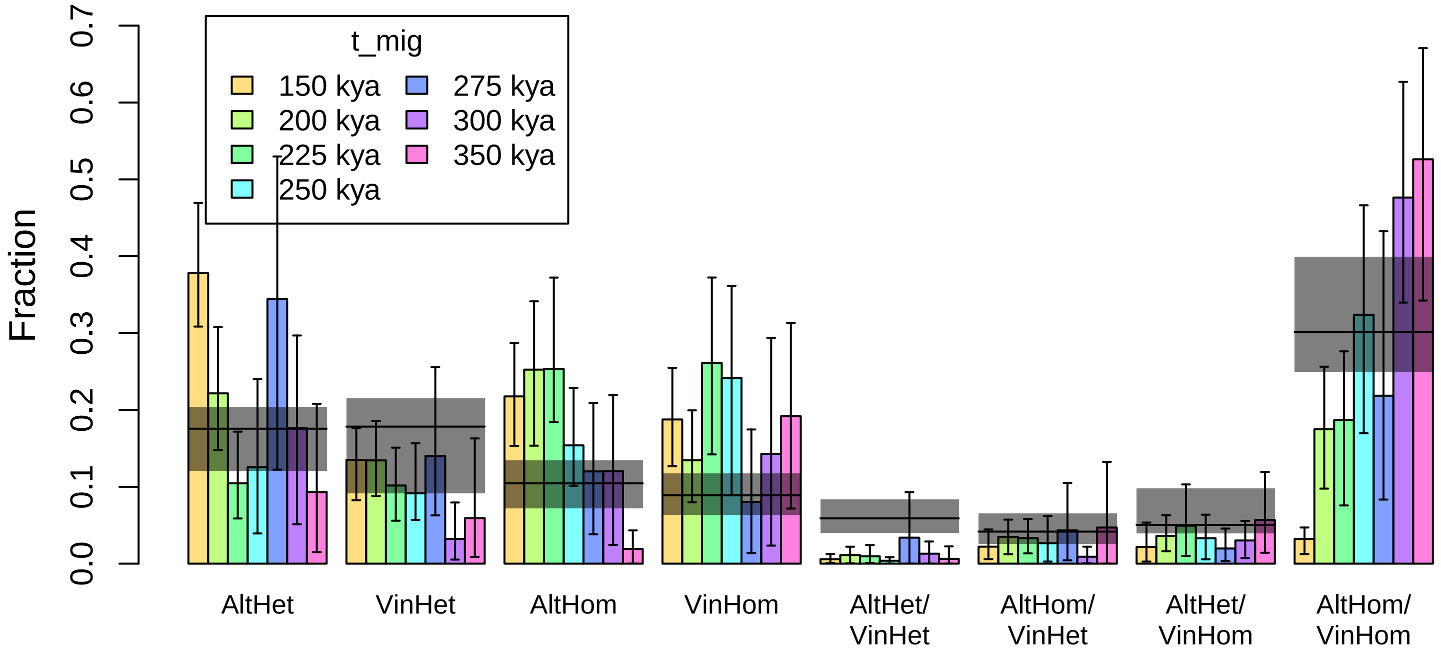

Figure S1-P: **Fig: Frequencies of Hum→Nea introgression categories, with $t_{mig} = 150$kya.** This is the same as Fig **??** in the main paper, but with all analysis performed using a model in which $t_{mig} = 150$kya rather than 250kya.

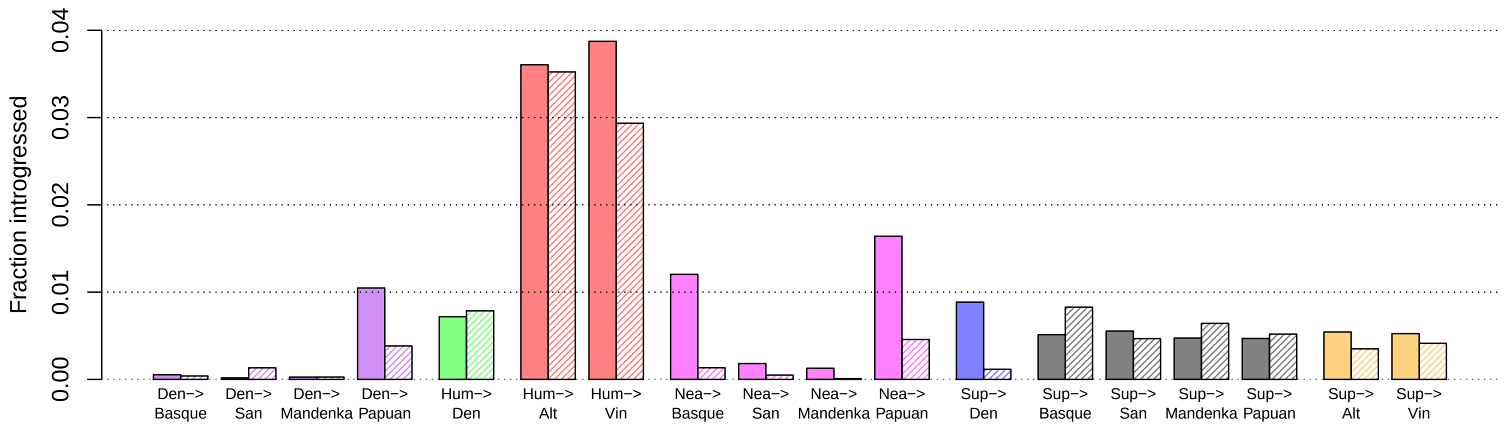

Figure S1-Q: **Fig: Coverages using out-of-Africa model and full population tree. Notation as in Fig S1-O, with the addition of Papuan and Basque individuals.**

Calculating the mutation rate map

We first extracted the hg19 (human), panTro4 (chimp), gorGor3 (gorilla), ponAbe2 (orangutan), and nomLeu3 (gibbon) sequences from the UCSC Genome Browser's 100-way vertebrate alignment. We then masked any segments of the alignment within 100bp of a phastCons element, using the union of all hg19 phastCons elements (phastConsElements46way, phastConsElements46wayPlacental, phastConsElements46wayPrimates, phastConsElements100way). We then ran phyloP on the alignment, using the options `--method LRT --features windows.bed --mode CONACC`, where the windows.bed file gives 100kb sliding windows of the human genome, staggered by 10kb. The substitution model used for the phyloP run was downloaded from `http://hgdownload.soe.ucsc.edu/goldenPath/hg19/phastCons100way/hg19.100way.phastCons.mod`. The phyloP run produced tree scaling factors for every 100kb window. These were converted to mutation rates by rescaling all factors to achieve a mean mutation rate of 1.45e-8/bp/generation. The mutation rate for a particular base was then mean of the mutation rates in the sliding windows which overlap that base. For substitution rates on chromosome X, we used the same procedure, with the exception that we used a substitution model specific to chromosome X, downloaded from `http://hgdownload.soe.ucsc.edu/goldenPath/hg19/phastCons46way/primates.chrX.mod`.

Runtime

ARGweaver-D is a computationally intensive method. For the analyses presented in this paper, we divided the genome into 2.2Mb windows, running each window on a single core. The expected runtime is dependent on the number of samples, number of populations, and the number and time of each migration event. Table S1-A gives a summary of total runtime for each genome-wide analysis for a variety of models. The runtime increases with the sample size, the number of migration bands, and also as the time of migration bands becomes more recent. While it is not practical to run ARGweaver-D genome-wide on a laptop computer, a genome-wide analysis can be done in a modest number of days with access to a multi-core server or cluster.

Table S1-A: **ARGweaver-D runtimes.**

**Description**	**Number lineages**	**Number pops**	**migrations**	**Run time (compute years)**
1 Papuan, 2 Africans, no super-archaic population, chimp	7	3	Sup→Hum @ 50kya	0.17
1 Papuan, 2 Africans, all archaics, no super-archaic, chimp	13	4	Nea→Hum @ 50kya<br>Den→Hum @ 50kya	0.59
1 Basque, 2 Africans, all archaics; no super-archaic, chimp	13	4	Nea→Hum @ 50kya<br>Den→Hum @ 50kya	0.79
2 Africans, all archaics, super-archaic ghost population, chimp	11	5	Hum→Nea @ 250kya<br>Hum→Den @ 250kya<br>Sup→Hum@ 250kya<br>Sup→Nea@ 250kya<br>Sup→Den@ 250kya	1.1
2 Africans, all archaics, super-archaic ghost population, chimp	11	5	Hum→Nea @ 250kya<br>Hum→Den @ 150kya<br>Sup→Hum@ 150kya<br>Sup→Nea@ 150kya<br>Sup→Den@ 150kya	1.4
8 Africans, all archaics, super-archaic ghost population, chimp	23	5	Hum→Nea @ 250kya<br>Hum→Den @ 250kya<br>Sup→Hum@ 250kya<br>Sup→Nea@ 250kya<br>Sup→Den@ 250kya	2.8
1 Basque, 1 Papuan, 2 Africans, all archaics, super-archaic ghost population, chimp	15	5	Nea→Hum @ 50kya<br>Den→Hum @ 50kya<br>Hum→Nea @ 250kya<br>Hum→Den @ 250kya<br>Sup→Hum@ 250kya<br>Sup→Nea@ 250kya<br>Sup→Den@ 250kya	4.6

This table gives total run-time; the genome was split into 1200 segments, each segment running in parallel on a single core. Not all rows respresent analyses that were presented in this paper. In some cases the analyses were only performed on a subset of the genome (but at least 40 segments), with total run-time extrapolated based on average time for each region. All runs included a single chimpanzee lineage. To the best of the author's memory, all runs were performed on Intel Xeon E5-2665@2.4Ghz processors. Memory requirements are modest (less than 1Gb/run).

References

1. Rasmussen MD, Hubisz MJ, Gronau I, Siepel A. Genome-wide inference of ancestral recombination graphs. PLoS Genet. 2014;10(5):e1004342.

2. Kelleher J, Etheridge AM, McVean G. Efficient coalescent simulation and genealogical analysis for large sample sizes. PLoS Comput Biol. 2016;12(5):1–22. doi:10.1371/journal.pcbi.1004842.

3. Kuhlwilm M, Gronau I, Hubisz MJ, de Filippo C, Prado-Martinez J, Kircher M, et al. Ancient gene flow from early modern humans into Eastern Neanderthals. Nature. 2016;530(7591):429–433.

4. Loh PR, Palamara PF, Price AL. Fast and accurate long-range phasing in a UK Biobank cohort. Nature Genetics. 2016;48:811–816.

5. O'Connell J, Gurdasani D, Delaneau O, Pirastu N, Ulivi S, Cocca M, et al. A general approach for haplotype phasing across the full spectrum of relatedness. PLOS Genetics. 2014;10(4):1–21. doi:10.1371/journal.pgen.1004234.

6. Browning SR, Browning BL. Rapid and accurate haplotype phasing and missing-data inference for whole-genome association studies by use of localized haplotype clustering. The American Journal of Human Genetics. 2007;81(5):1084–1097. doi:10.1086/521987.

7. V Barroso G, Puzovi N, Dutheil JY. Inference of recombination maps from a single pair of genomes and its application to ancient samples. PLOS Genetics. 2019;15(11):1–21. doi:10.1371/journal.pgen.1008449.

8. Hinch AG, Tandon A, Patterson N, Song Y, Rohland N, Palmer CD, et al. The landscape of recombination in African Americans. Nature. 2011;476(7359):170–175.

9. Li H, Durbin R. Inference of human population history from individual whole-genome sequences. Nature. 2011;475:493–496.

10. Schiffels S, Durbin R. Inferring human population size and separation history from multiple genome sequences. Nat Genet. 2014;46(8):919–925.

11. Gronau I, Hubisz MJ, Gulko B, Danko CG, Siepel A. Bayesian inference of ancient human demography from individual genome sequences. Nat Genet. 2011;43(10):1031–1034.

12. Terhorst J, Kamm JA, Song YS. Robust and scalable inference of population history from hundreds of unphased whole genomes. Nature Genetics. 2017;49(2):303–309. doi:10.1038/ng.3748.

13. Prüfer K, Racimo F, Patterson N, Jay F, Sankararaman S, Sawyer S, et al. The complete genome sequence of a Neanderthal from the Altai Mountains. Nature. 2014;505(7481):43–49.

14. Prüfer K, de Filippo C, Grote S, Mafessoni F, Korlević P, Hajdinjak M, et al. A high-coverage Neandertal genome from Vindija Cave in Croatia. Science. 2017;358(6363):655–658. doi:10.1126/science.aao1887.

15. Sankararaman S, Mallick S, Patterson N, Reich D. The combined landscape of Denisovan and Neanderthal ancestry in present-day humans. Current Biology. 2016;26:1241–1247.
