# Supplementary material for: Mapping gene flow between ancient hominins through demography-aware inference of the ancestral recombination graph
Source: PLoS Genet. 2020 Aug 6;16(8):e1008895. doi: 10.1371/journal.pgen.1008895 (PMC7410169; doi:10.1371/journal.pgen.1008895)
Supplement: S1 Table — (PDF) [file pgen.1008895.s002.pdf]

S1 Table. Sup→Den regions overlapping Den→Hum regions predicted by the CRF

Location (hg19)	count	overlapping genes
chr15:56880301-56943860	10	RP11-1129I3.1, ZNF280D
chr5:35268551-35472820	9	U3
chr15:79936231-80045380	7	
chr2:183978771-184038340	7	NUP35
chr1:40622111-40751801	6	RLF, RNU6-1237P, TMCO2, RP1-39G22.7, ZMPSTE24
chr4:143486431-143606100	6	INPP4B, RP11-223C24.1
chr15:63493991-63599658	5	RAB8B, APH1B
chr17:30992260-31232970	3	MYO1D, RP11-220C2.1, Y_RNA, AC084809.2, AC084809.3
chr5:74577091-74897550	3	CTD-2235C13.2, HMGCR, COL4A3BP, CTD-2235C13.3, POLK, RNU7-175P, CTC-366B18.2
chr20:18369011-18456230	3	DZANK1, RNA5SP476, POLR3F, MIR3192
chr2:104441221-104575299	2	AC013727.1, AC013727.2, RP11-76I14.1
chr3:156394341-156515810	2	TIPARP, RP11-392A22.2
chr8:97918711-98192640	2	CPQ, KB-1958F4.2, KB-1958F4.1
chr8:56673021-56798570	2	TMEM68, TGS1, LYN
chr13:77606499-77899730	1	MYCBP2, MYCBP2-AS1, RP11-226E21.2
chr4:85723291-85798820	1	WDFY3, RP11-147K21.1
chr6:131062559-131237440	1	SMLR1, EPB41L2
chr7:83335141-83452959	1	
chr10:52582401-52700350	1	A1CF, RP11-449O16.2
chr3:129951121-130100515	1	COL6A5, AC093004.1

The “count” column shows the number of non-African SGDP individuals who have Denisovan introgression at this locus. We restricted this list to Sup→Den regions for which at least 90% of SGDP individuals without Denisovan introgression have a higher divergence to the Denisovan than to Neanderthals.
