# Supplementary material for: Mapping gene flow between ancient hominins through demography-aware inference of the ancestral recombination graph
Source: PLoS Genet. 2020 Aug 6;16(8):e1008895. doi: 10.1371/journal.pgen.1008895 (PMC7410169; doi:10.1371/journal.pgen.1008895)
Supplement: S2 Table — (PDF) [file pgen.1008895.s003.pdf]

S2 Table. Sup→Nea regions overlapping Nea→Hum regions predicted by the CRF

Location (hg19)	count	overlapping genes
chr6:8450001-8563749	71	HULC
chr7:44396121-44543978	37	RP5-844F9.1, NUDCD3, RNU6-1097P
chr4:121531631-121587672	32	RP11-501E14.1
chr9:30239959-30438940	21	LINC01242
chr7:50432351-50497990	17	IKZF1, CTC-736O2.1
chr9:94891421-95445440	17	snoU13, RP11-62C3.6, IARS, SNORA84, NOL8, CENPP, OGN, OMD, ASPN, ECM2, MIR4670, IPPK
chr3:16970431-17045770	15	PLCL2, MIR3714
chr6:120748701-120851630	14	RNU6-214P
chr7:85753641-85880460	14	
chr9:73603471-73725370	13	TRPM3
chr11:42691811-42766780	13	
chr4:106603601-106693390	9	INTS12, GSTCD, RP11-45L9.1
chr15:67472779-67650950	8	SMAD3, AAGAB, IQCH
chr6:41014213-41153400	7	APOBEC2, OARD1, NFYA, TREML1, TREM2
chr2:84214131-84279410	7	
chr4:42929991-43023170	5	GRXCR1
chr4:161759472-162023170	5	AC106860.1
chr5:342721-451430	5	AHRR, C5orf55, EXOC3
chr9:88250001-88377170	4	AGTPBP1, RP11-202I11.2
chr4:117545891-117601428	4	
chr4:18307191-18446628	3	
chr12:92114981-92187270	3	
chr4:18307191-18368540	3	
chr6:46356781-46434892	3	RCAN2
chr18:47574231-47700390	2	MYO5B
chr4:81260001-81631350	2	C4orf22
chr6:44893490-45311660	2	SUPT3H, MIR586, RUNX2
chr13:84262861-84384570	1	
chr13:87243951-87386382	1	
chr3:100416521-100565100	1	TFG, ABI3BP
chr4:18084421-18196990	1	
chr4:98345401-98553210	1	RP11-18N21.2, RP11-681L8.1, AC034154.1, STPG2
chr13:65910681-66015839	1	

The “count” column shows the number of non-African SGDP individuals who have Neanderthal introgression at this locus. We restricted this list to Sup→Nea regions for which at least 90% of SGDP individuals without Neanderthal introgression have a higher divergence to the Neanderthal than to Denisovans.
