# Supplementary figures and images for: Mapping gene flow between ancient hominins through demography-aware inference of the ancestral recombination graph
Source: PLoS Genet. 2020 Aug 6;16(8):e1008895. doi: 10.1371/journal.pgen.1008895 (PMC7410169; doi:10.1371/journal.pgen.1008895)

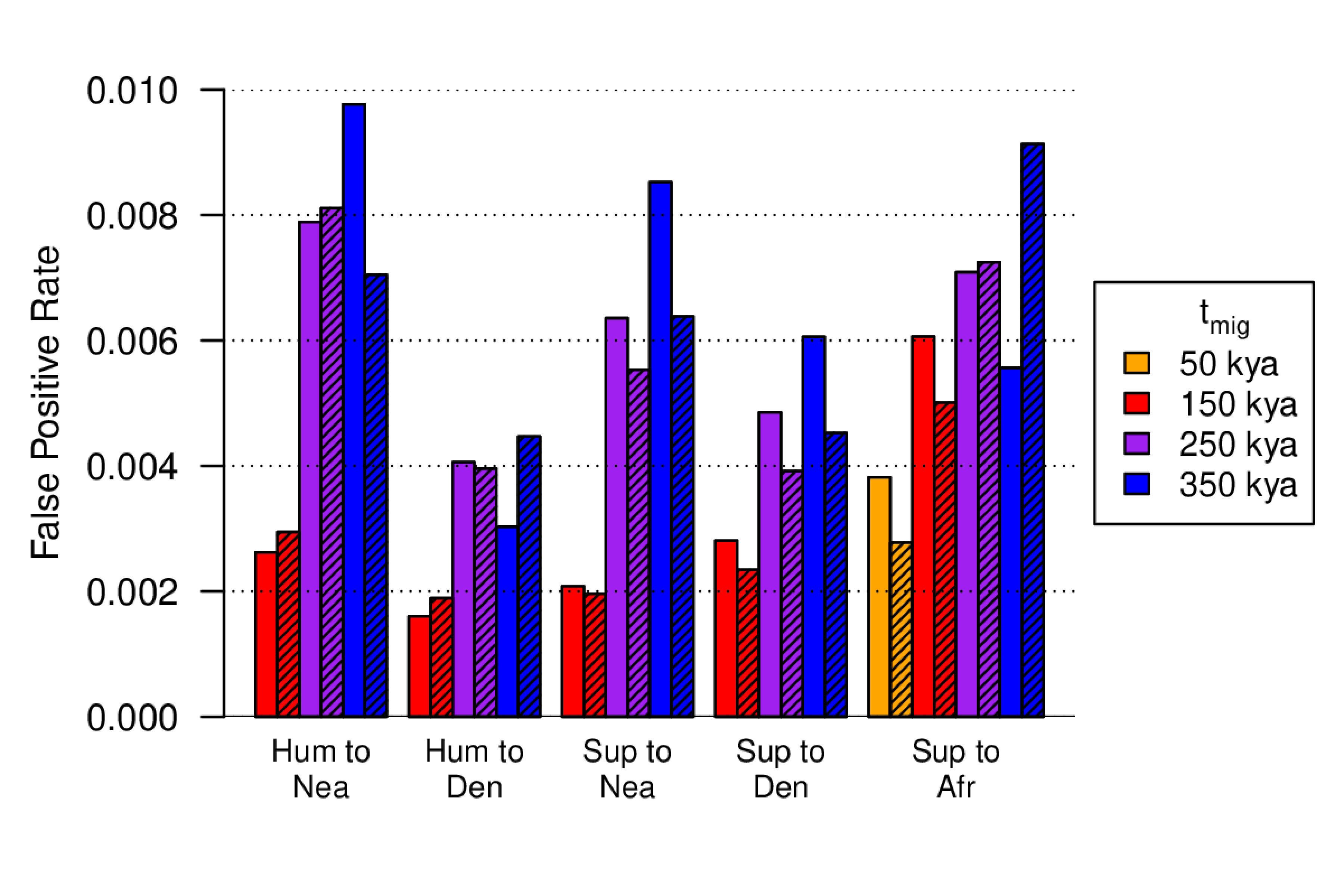

Supplement: S1 Fig — Color indicates the value of tmig. Shaded bars have tdiv = 1.5Mya and solid bars have tdiv = 1.0Mya. False positive rates are calculated base-wise using a posterior probability cut-off of 0.5. The same set of underlying data was used for all the calculations in this plot; it was simulated as in Fig 3, but with no true migration events. (TIF) [file pgen.1008895.s004.tif]

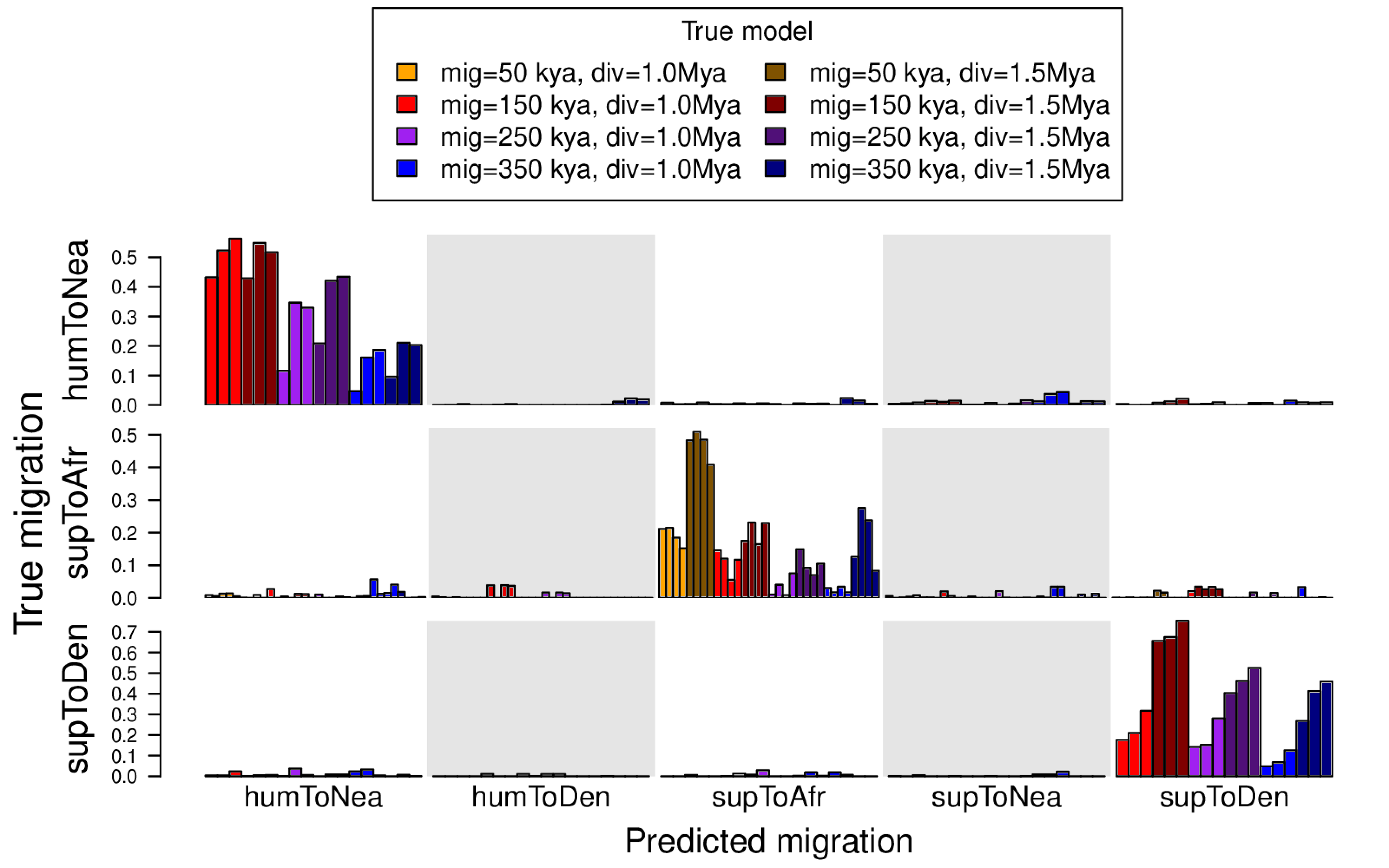

Supplement: S2 Fig — Each row shows a true migration category, and each column shows the fraction of bases predicted in the category indicated at the foot of the column. The color of each bar represents the true parameters used in simulation, as indicated in the legend, with darker colors used for the older super-archaic divergence time. Multiple bars of the same color show results on the same data set, using an ARGweaver-D model with a different tmig. The value of tmig used by ARGweaver-D is not indicated in the plot, but increases from left-to-right: tmig = 50, 150, 250, 350kya, with 50kya only shown for Sup→Afr. All the models used tdiv = 1Mya; the plot with tdiv = 1.5Mya is nearly identical. (TIF) [file pgen.1008895.s005.tif]

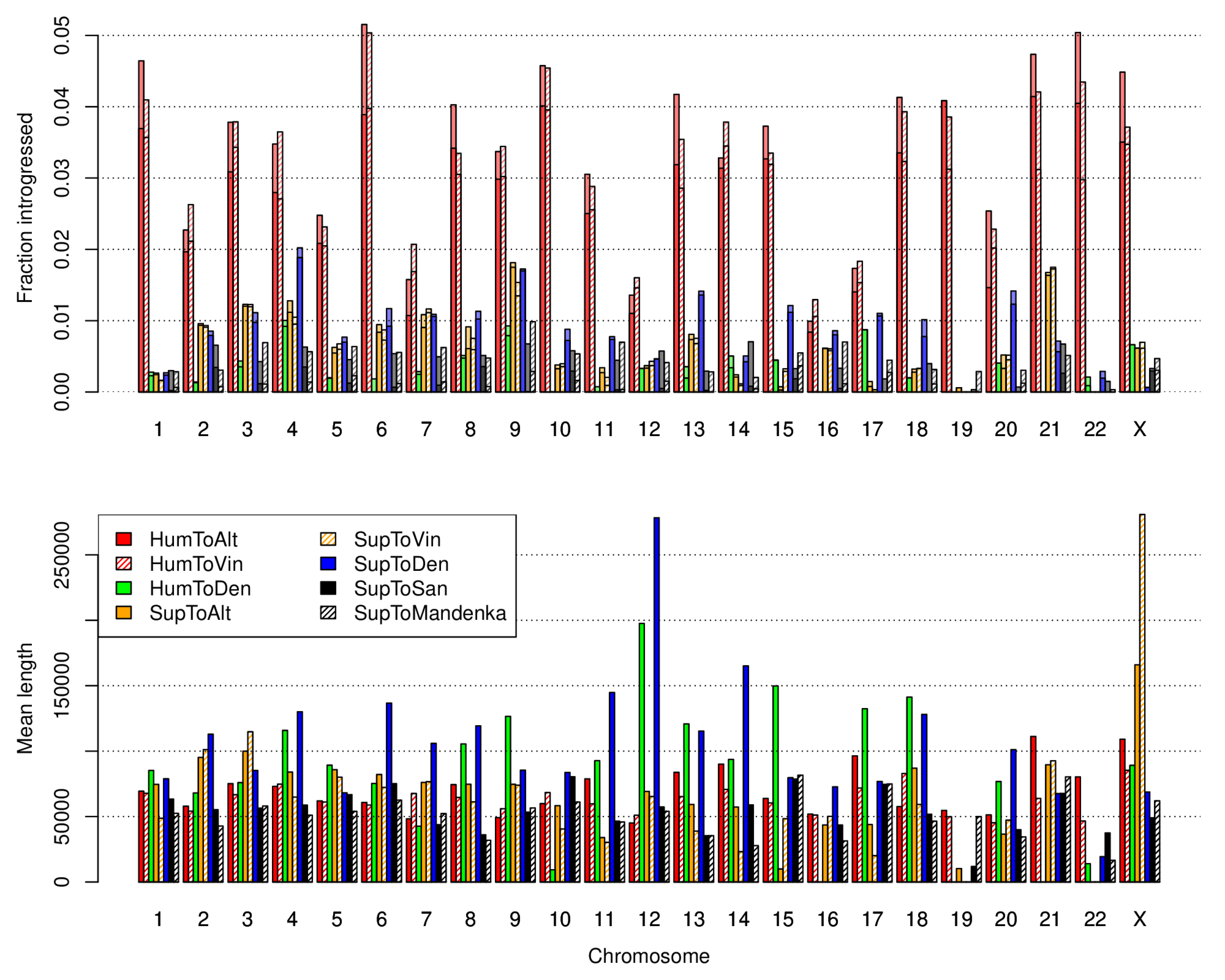

Supplement: S3 Fig — The top plot shows average coverage of predicted introgressed regions per haploid genome, with darker portions representing homozygous regions. The bottom shows average length of introgressed regions by chromosome. (TIF) [file pgen.1008895.s006.tif]

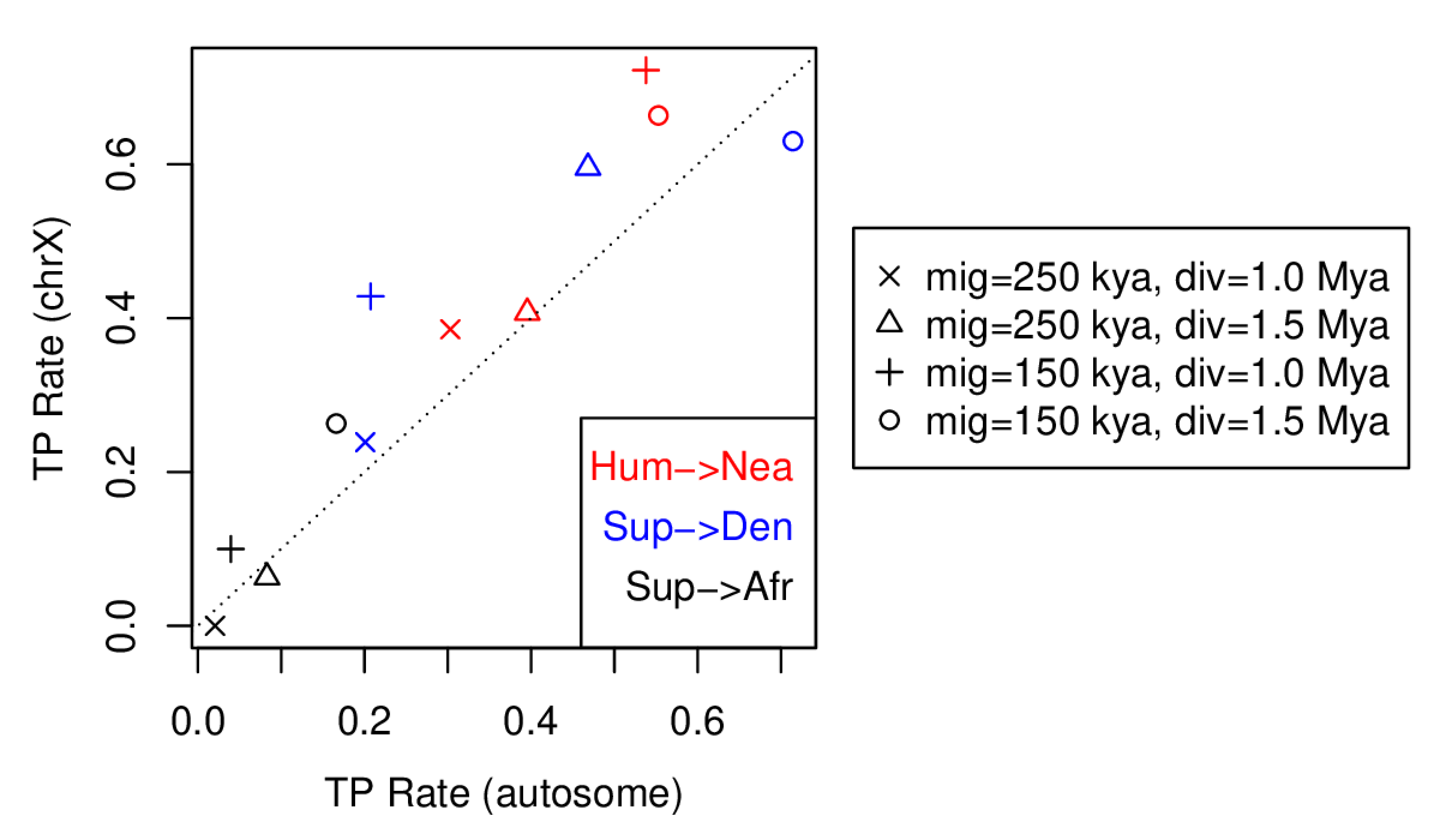

Supplement: S4 Fig — The y-axis shows true positive rates from simulations where population sizes were multiplied by 0.75 to roughly approximate X chromosome demography. Different plotting characters are used for different simulation models, as indicated in the legend. All ARGweaver-D analysis was done with tmig = 250kya and tdiv = 1.0Mya. (TIF) [file pgen.1008895.s007.tif]

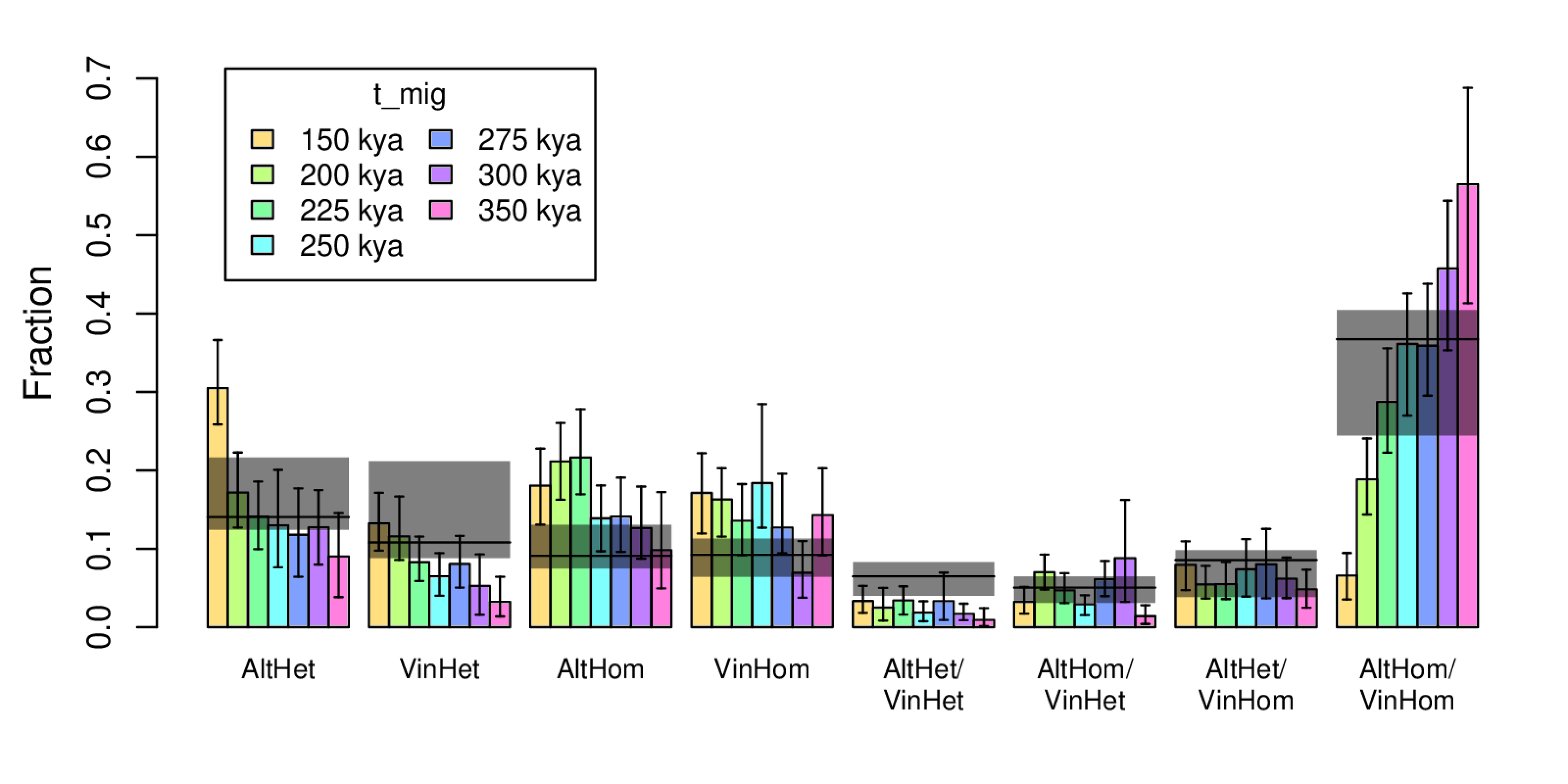

Supplement: S5 Fig — For both the real and simulated data, Hum→Nea regions were ascertained with ARGweaver-D using a model with tmig = 250kya and tdiv = 1Mya. These regions were classified as heterozygous/homozyogus in the Altai Neanderthal (AltHet/AltHom), and in the Vindija Neanderthal (VinHet/VinHom), depending on which branches are in the migrant state in the majority of sampled ARGs. Here, the colored bars represent the fraction of Hum→Nea bases in each category for simulated data sets generated with different values of tmig; the error bars show 95% confidence intervals (CIs) computed using 100 bootstrap replicates across the introgressed elements. The horizontal black lines represent the amount observed in the real data, with the gray boxes showing the CIs, also obtained by the same bootstrap process. For this figure, long homozygous stretches of the archaic genomes annotated in [9] were excluded. (TIF) [file pgen.1008895.s008.tif]

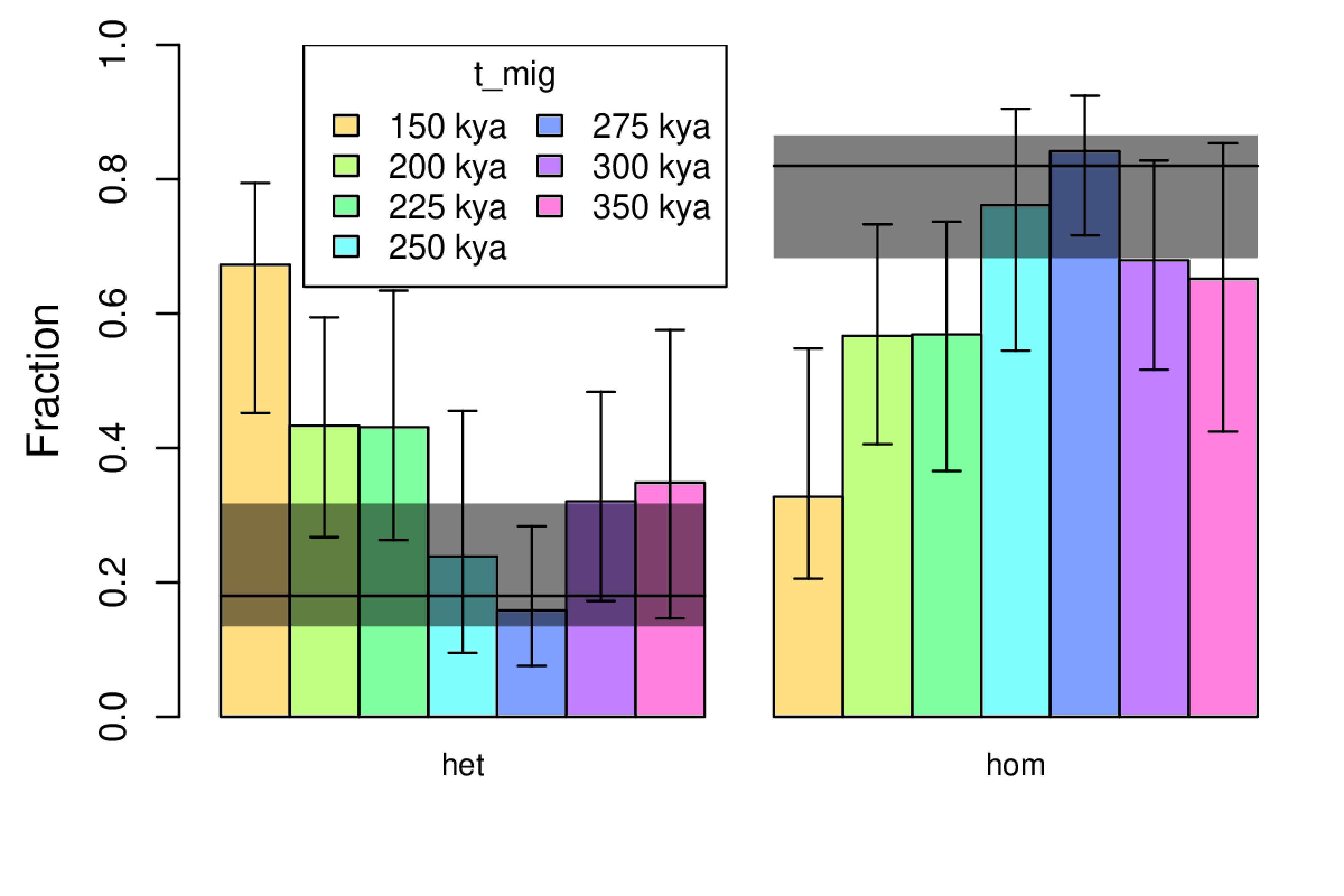

Supplement: S6 Fig — This figure is analygous to S5 Fig; here we look at putative Sup→Den regions. Because there is only one Denisovan individual, there are only two categories: heterozygous or homozygous. Note that while we expect rates of heterozygosity to decrease with migration time, the confidence intervals here are wide, as the power to detect old events is very low. As in S5 Fig, long homozygous stretches of the Denisovan genome annotated by [9] were excluded. (TIF) [file pgen.1008895.s009.tif]

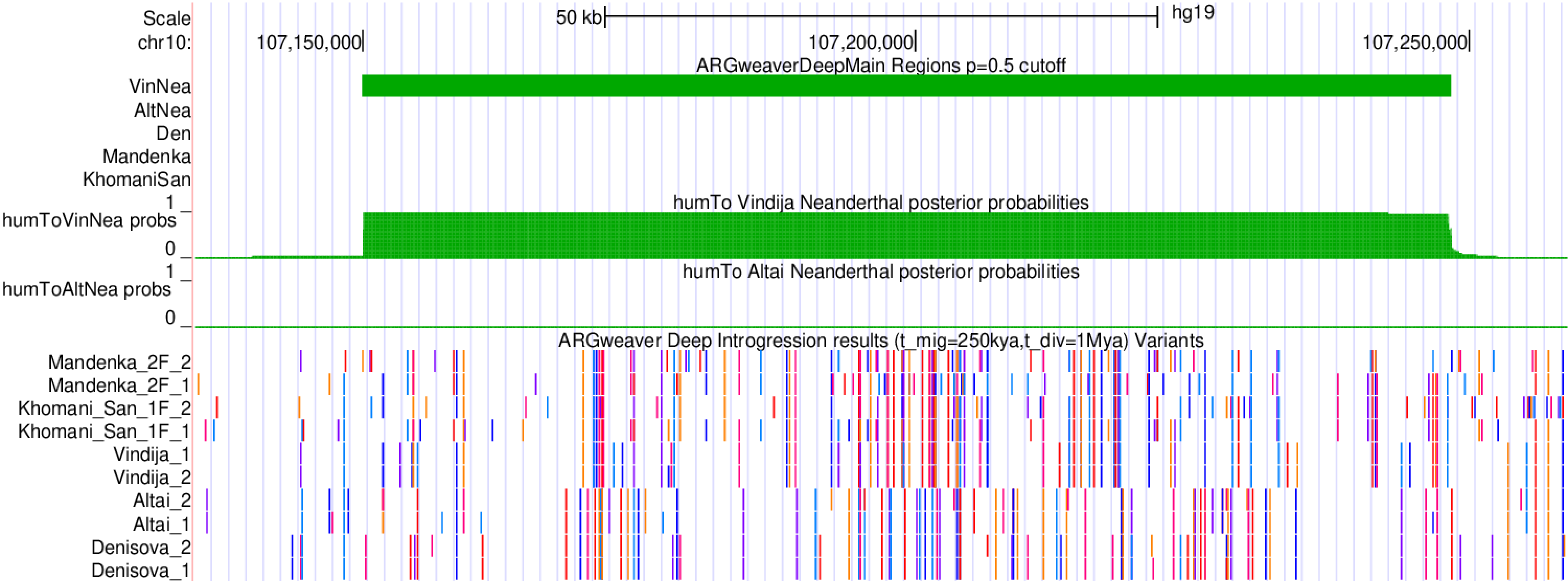

Supplement: S7 Fig — This region on chromosome 10 has a high-probability introgressed region in both Vindija (but neither Altai) haplotypes. The top green bar indicates a predicted Hum→Nea region in Vindija, and below this is the posterior probability of introgression across the region in both Neanderthals. The variant track is similar to Fig 8. Here, we see almost identical haplotypes between Vindija and the Africans, whereas Altai shares haplotypes with the Denisovan. (TIF) [file pgen.1008895.s010.tif]

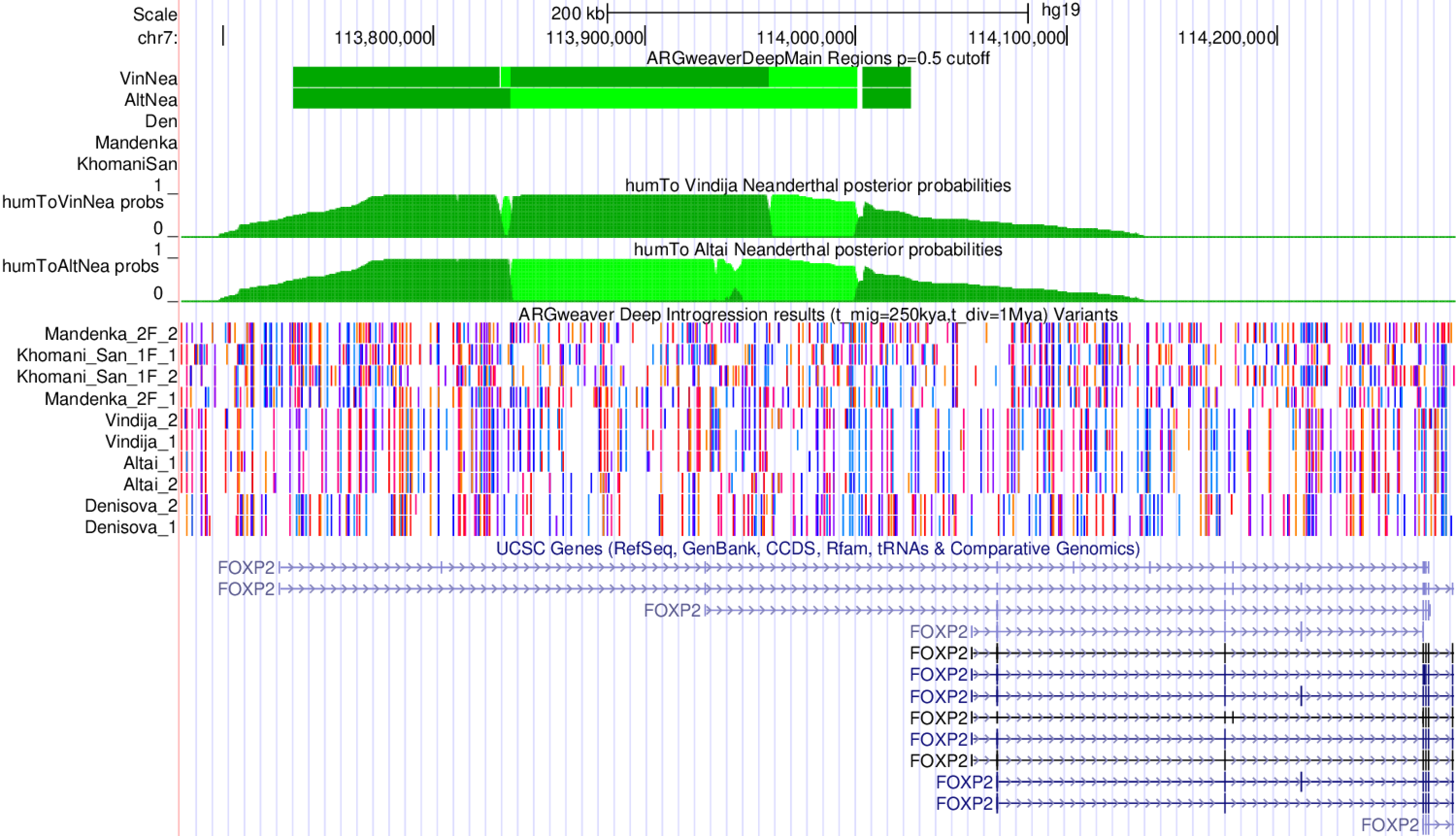

Supplement: S8 Fig — Exon 7, which contains human-chimp substitutions shared by Neanderthals that may be involved with human speech, is located at the very right of this plot, and is not predicted introgressed. As in Fig 7, the light green implies heterozygous Hum→Nea introgression, whereas dark green is homozygous. (TIF) [file pgen.1008895.s011.tif]

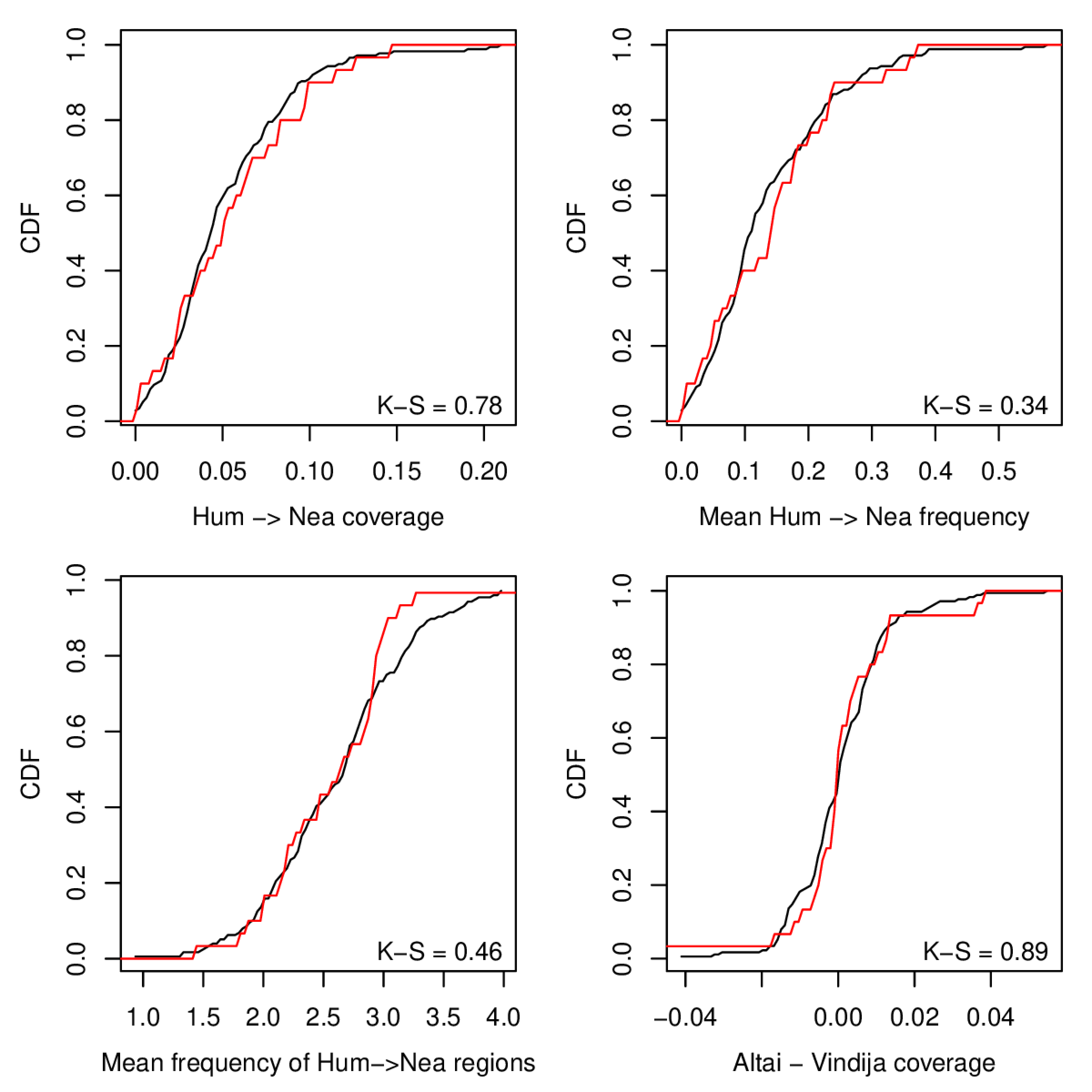

Supplement: S9 Fig — We compared the distribution of various statistics across all non-overlapping 15Mb windows in the genome (black), to the distribution within deserts of Neanderthal introgression in humans of at least 10Mb (red). We excluded any window that crosses a telomere or centromere, or where ≥ 50% of the window does not pass our filters. In the bottom-right corner of each plot is shown the Kolmogorov-Smirnov statistic p-value, indicating that there is no significant difference between the black and red distributions. The statistics shown are indicated on the x-axis label. “Hum→Nea coverage” is average fraction of the window that contains any Hum→Nea region. “Mean Hum→Nea frequency” is the average number of introgressed haploid lineages of Hum→Nea across the window (where a frequency of zero indicates no introgression, and a frequency of 4 indicates homozygous introgression in Altai and Vindija). “Mean frequency of Hum→Nea regions” is the mean frequency, among regions with Hum→Nea calls. “Altai—Vindija coverage” is difference in mean coverage between the Altai and Vindija within each window. (TIF) [file pgen.1008895.s012.tif]
